# Data-Dependent Conditional Priors for Unsupervised Learning of Multimodal Data †

**DOI:** 10.3390/e22080888

**Published:** 2020-08-13

**Authors:** Frantzeska Lavda, Magda Gregorová, Alexandros Kalousis

**Affiliations:** 1 Faculty of Science, Computer Science Department, University of Geneva, 1214 Geneva, Switzerland; 2 Geneva School of Business Administration (DMML Group), HES-SO, 1227 Geneva, Switzerland; magda.gregorova@hesge.ch (M.G.); alexandros.kalousis@hesge.ch (A.K.)

**Keywords:** VAE, generative models, learned prior

## Abstract

One of the major shortcomings of variational autoencoders is the inability to produce generations from the individual modalities of data originating from mixture distributions. This is primarily due to the use of a simple isotropic Gaussian as the prior for the latent code in the ancestral sampling procedure for data generations. In this paper, we propose a novel formulation of variational autoencoders, conditional prior VAE (CP-VAE), with a two-level generative process for the observed data where continuous z and a discrete c variables are introduced in addition to the observed variables x. By learning data-dependent conditional priors, the new variational objective naturally encourages a better match between the posterior and prior conditionals, and the learning of the latent categories encoding the major source of variation of the original data in an unsupervised manner. Through sampling continuous latent code from the data-dependent conditional priors, we are able to generate new samples from the individual mixture components corresponding, to the multimodal structure over the original data. Moreover, we unify and analyse our objective under different independence assumptions for the joint distribution of the continuous and discrete latent variables. We provide an empirical evaluation on one synthetic dataset and three image datasets, FashionMNIST, MNIST, and Omniglot, illustrating the generative performance of our new model comparing to multiple baselines.

## 1. Introduction

Variational autoencoders (VAEs) [1,2] are deep generative models for learning complex data distributions. They consist of an encoding and decoding network parametrizing the variational approximate posterior and the conditional data distributions in a latent variable generative model.

Though powerful and theoretically elegant, the VAEs in their basic form suffer from multiple deficiencies that stem from the mathematically convenient yet simplistic distributional assumptions. Multiple strategies have been proposed to increase the richness or interpretability of the latent code [3,4,5,6,7,8,9,10,11,12]. These mostly argue for more flexible posterior inference procedure or for the use of more complex approximate posterior distributions to facilitate the encoding of non-trivial data structures within the latent space.

In this paper, we reason that for generating realistic samples of data originating from complex distributions, it is the prior that lacks expressiveness. Accordingly, we propose a new VAE formulation, conditional prior VAE (CP-VAE), with two-level hierarchical generative model combining categorical and continuous (Gaussian) latent variables.

The hierarchical conditioning of the continuous latent variable on the discrete latent component is particularly suitable for modelling multimodal data distributions, such as distributional mixtures. Importantly, it also gives us better control of the procedure for generating new samples. Unlike in the standard VAE, we can sample data from specific mixture components at will. This is particularly critical if the generative power of VAEs shall be used in conjunction with methods requiring the identification of the distributional components, such as in continual learning [13,14].

As recently shown [12,15], without supervision (as in our setting), enforcing independence factorization in the latent space does not guarantee recovering meaningful sources of variation in the original space. Therefore, in our CP-VAE formulation, we let the model fully utilize the capacity of the latent space by allowing for natural conditional decomposition in the generative and inference graphical models.

We formulate the corresponding variational lower bound on the data log-likelihood and use it as the optimization objective in the training. In the spirit of empirical Bayes, we propose estimating the parameters of the conditional priors from the data together with the parameters of the variational posteriors in a joint learning procedure. This ensures that the inferred structure of the latent space can be exploited in data generations.

## 2. From Variational Inference (VI) Objective to VAE Objective

Variational autoencoders (VAEs) [1,2] are deep Bayesian generative models that rely on the principals of amortized variational inference to approximate the complex distributions p(x) from which the observed data $S={\left\{x_{i}\right\}}_{i=1}^{N}$ originate.

In their basic form, they model the unknown ground-truth p(x) by a parametric distribution $p_{\theta }\left(x\right)$ with a latent variable generative process

(1) $$p_{\theta }\left(x\right)=\int p_{\theta }\left(x|z\right)p\left(z\right)dz\;.$$

Computing $p_{\theta }\left(x\right)$ is difficult and usually turns out to be an intractable distribution. However, we can learn a surrogate loss to the original likelihood $p_{\theta }\left(x\right)$ while using Variational Inference principles.

### 2.1. Variational Inference

Variational Inference involves the optimization of an approximation to the intractable posterior. In Variational Inference, we specify a family of tractable distributions $q_{\phi }\left(z|x\right)$. The goal is to find the best variational parameters ϕ, such that the approximation $q_{\phi }\left(z|x\right)$ is as close as possible to the intractable posterior $p_{\theta }\left(z|x\right)$, i.e., $q_{\phi }\left(z|x\right)∼p_{\theta }\left(z|x\right)$. We do that by minimizing the KL divergence of the approximation $q_{\phi }\left(z|x\right)$ from the true posterior $p_{\theta }\left(z|x\right)$,

(2) $$\phi^{*}=argmin_{\phi }\mathrm{KL}\left(q_{\phi }\left(z|x\right)∥p_{\theta }\left(z|x\right)\right)$$

where the KL divergence is equal to:

(3) $$\begin{array}{cc} \mathrm{KL}\left(q_{\phi }\left(z|x\right)∥p_{\theta }\left(z|x\right)\right)\; & =E_{q_{\phi }\left(z|x\right)}\left[\log\frac{q_{\phi }\left(z|x\right)}{p_{\theta }\left(z|x\right)}\right] \end{array}$$

Reordering the terms of Equation (3), we have:

(4) $$\begin{array}{cc} \log p_{\theta }\left(x\right) & =\underset{KL}{\underset{︸}{\mathrm{KL}\left(q_{\phi }\left(z|x\right)∥p_{\theta }\left(z|x\right)\right)}}+\underset{ELBO}{\underset{︸}{E_{q_{\phi }\left(z|x\right)}\left[\log p_{\theta }\left(z,x\right)-\log q_{\phi }\left(z|x\right)\right]}}\\ \; & =\mathrm{KL}\left(q_{\phi }\left(z|x\right)∥p_{\theta }\left(z|x\right)\right)+L\left(x;\theta ,\phi \right) \end{array}$$

The first term is the initial KL divergence we want to minimize in VI. Since the Kullback-Leibler divergence is always greater than or equal to zero, the second term L, called the Evidence Lower BOund, ELBO, is the variational lower bound on the marginal log likelihood $\log p_{\theta }\left(x\right)$. The closer $\mathrm{KL}\left(q_{\phi }\left(z|x\right)∥p_{\theta }\left(z|x\right)\right)$ is to 0, the closer L(x;θ,ϕ) will be to logp(x).

(5) $$\log p_{\theta }\left(x\right)\geq L\left(x;\theta ,\phi \right)$$

### 2.2. Variational Autoencoders

In VAEs, the typical assumptions are of a simple isotropic Gaussian prior p(z) for the latent variable z and, depending on the nature of the data x, factorized Bernoulli or Gaussian distributions for the data conditionals $p_{\theta }\left(x|z\right)$. These per-sample conditionals are parametrized by a deep neural network, a decoder. Once the decoder network is properly trained, we can sample new data examples from the learned data distribution $p_{\theta }\left(x\right)$ by ancestral sampling procedure: sample the latent z from the prior p(z) and pass it through the stochastic decoder $p_{\theta }\left(x|z\right)$ to obtain the sample x.

The VAEs employ the strategy of amortized variational inference. They approximate the intractable posteriors $p_{\theta }\left(z|x\right)$ by factorized Gaussian distributions $q_{\phi }\left(z|x\right)$ and infer the variational parameters ϕ of the approximate per-sample posteriors through a deep neural network, an encoder.

The encoder and decoder networks are trained end-to-end by stochastic gradient-based optimization maximizing the sample estimate of The Evidence Lower Bound $L_{\theta ,\phi }=E_{p(x)}L_{\theta ,\phi }\left(x\right)$ on the data log-likelihood.

$$\begin{array}{c} \frac{1}{N}\sum_{i}^{N}\log p_{\theta }\left(x_{i}\right)\geq \frac{1}{N}\sum_{i}^{N}L_{\theta ,\phi }\left(x_{i}\right)\approx L_{\theta ,\phi } \end{array}$$

(6) $$\begin{array}{c} \;L_{\theta ,\phi }\left(x\right)=\underset{A}{\underset{︸}{E_{q_{\phi }\left(z|x\right)}\log p_{\theta }\left(x|z\right)}}-\underset{B}{\underset{︸}{\mathrm{KL}\left(q_{\phi }\left(z|x\right)\left|\right|p\left(z\right)\right)}}\; \end{array}$$

The first term *A* in Equation (6) can be seen as a negative reconstruction cost, term *B* penalizes the deviations of the approximate posterior from the fixed prior p(z) and it has a regularizing effect on the model learning. The term *A* encourages the latent variable z to contain meaningful information in order to reconstruct *x* and at the same time, the term *B* penalizes the approximate posterior for deviating from the prior, preventing the model from simply memorizing each data point.

The gradients of the lower bound with respect to the model parameters θ can be obtained streighforwardly through Monte Carlo estimation. For the posterior parameters ϕ, the gradients are estimated by stochastic backpropagation while using a location-scale transformation known as the reparametrization trick.

### 2.3. Posterior Collapse and Mismatch between the True and the Approximate Posterior

In Equation (4), we see that, in order to improve the variational lower bound, the approximate posterior $q_{\phi }\left(z|x\right)$ should match the true posterior $p_{\theta }\left(z|x\right)$. In other words, the ELBO is tight when $q_{\phi }\left(z|x\right)=p_{\theta }\left(z|x\right)$. As we mentioned above, the choice of $q_{\phi }\left(z|x\right)$ is often a factorized Gaussian distribution for simplicity and efficiency. In this way, the approximate posterior is simplified and it is hard for it to match the possible complex true posterior.

Moreover, by minimizing the KL-term in Equation (6), we encourage the approximate posterior to be close to the simple isotropic Gaussian prior p(z), an even simpler distribution. This may cause the main issue with VAE, called posterior collapse, where the model learns to ignore the latent variable and the approximate posterior mimics the prior, $q_{\phi }\left(z|x\right)\approx p\left(z\right)$ [6,16]. This reduces the capacity of the generative model, making it impossible for the decoder to use all the information of all of the latent dimensions or even not use, at all, the latent variable. This problem is more common when the decoder $p_{\theta }\left(x|z\right)$ is parametrised as an autoregressive model [6].

The posterior collapse and, consequently, the mismatch between the true and the approximate posterior motivates a direct improvement of variational inference by assuming/learning a more flexible posterior approximation for variational inference [3,5], or an indirect improvement assuming a more flexible prior [6,17]. Moreover a range of heuristic approaches in the literature have attempted to diminish the effect of the KL term in the ELBO to alleviate posterior collapse [18], or propose new regularizers [9].

### 2.4. Optimal Prior

Even though the prior in the VAEs is usually modelled by a simple isotropic Gaussian distribution, this assumption is a source of over-regularization, and is one of the causes of the poor density estimation performance [19].

To derive the optimal prior, we reformulate the VAE objective Equation (4). By maximizing the ELBO, we force the approximate posterior to be close to the true one and the marginal likelihood $p_{\theta }\left(x\right)$ to be close to the data distribution, $p_{D}\left(x\right)$, as we see in Equation (7).

(7) $$\begin{array}{cc} L_{\theta ,\phi }\; & =E_{p_{D}\left(x\right)}\left[\log p_{\theta }\left(x\right)-\mathrm{KL}\left(q_{\phi }\left(z|x\right)∥p_{\theta }\left(z|x\right)\right)\right]\\ \; & =E_{p_{D}\left(x\right)}\left[\log\frac{p_{\theta }\left(x\right)}{p_{D}\left(x\right)}p_{D}\left(x\right)\right]-E_{p_{D}\left(x\right)}\left[\mathrm{KL}(q_{\phi }\left(z|x\right)∥p_{\theta }\left(z|x\right))\right]\\ \; & =-\mathrm{KL}\left(p_{D}\left(x\right)∥p_{\theta }\left(x\right)\right)-H\left(p_{D}\left(x\right)\right)-E_{p_{D}\left(x\right)}\left[\mathrm{KL}(q_{\phi }\left(z|x\right)∥p_{\theta }\left(z|x\right))\right] \end{array}$$

The maximizing solution is equal to the negative entropy of the data distribution, $-H(p_{D}\left(x\right))$ and it is reached when the two KL divergence terms are equal to zero, meaning that the approximate posterior becomes equal to the true one, $q_{\phi }\left(z|x\right)=p_{\theta }\left(z|x\right)$ and the data distribution equal to the true distribution, $p_{D}\left(x\right)=p_{\theta }\left(x\right)$.

In this optimal case, the marginal approximate posterior $q_{\phi }\left(z\right)$ matches the prior, $q_{\phi }\left(z\right)=\int_{x}q_{\phi }\left(z|x\right)p_{D}\left(x\right)dx=\int_{x}p_{\theta }\left(z|x\right)p_{\theta }\left(x\right)dx=p\left(z\right)$. This indicates that the optimal prior for maximizing the ELBO is the marginal approximate posterior.

$$\begin{array}{c} p\left(z\right)\leftarrow q_{\phi }\left(z\right)=\frac{1}{N}\sum_{i=1}^{N}q_{\phi }\left(z|x_{i}\right) \end{array}$$

where the summation is performed over all training samples $x_{i};\;i=1,\cdots ,N$. The marginal posterior is the average of the approximate posterior with as many components as data points in the sample *S*, and it can been seen as Mixture of Gaussians (MoG) over all the data. However, this extreme case leads to over-fitting as this prior essentially memorizes the training set. Moreover, it is computationally inefficient, since it is very expensive to compute at every training iteration.

A natural approximation of the marginal approximate posterior prior can be a Mixture of Gaussian (MoG) prior in a random subset of the data, $p\left(z\right)=\frac{1}{K}\sum_{k=1}^{K}p_{\phi }\left(z|x_{k}\right)$ with K<N components.

Alternatively, marginal approximate posterior can be modelled while using a mixture of posteriors over learned virtual observations (pseudo-inputs) with a fixed number of components $p\left(z\right)≃\frac{1}{K}\sum_{k}q_{\phi }\left(z|u^{\left(k\right)}\right)$ [17]. Hence, the original standard Gaussian prior is replaced by a flexible multi-modal distribution.

## 3. Related Work

Since their introduction in 2014 [1,2], variational autoencoders have become one of the major workhorses for large-scale density estimation and unsupervised representation learning. Multitudes of variations on and enhancements of the original design have been proposed in the literature. These can broadly be categorized into four large groups (with significant overlaps as many methods mix multiple ideas to achieve the best possible performance).

First, it has been argued that optimizing the variational bound Equation (6) instead of the intractable likelihood $p_{\theta }\left(x\right)$ inhibits the VAEs to learn useful latent representations for both data reconstructions and downstream tasks. Methods using alternative objectives aim to encourage the learning towards representations that are better aligned with the data (measured by mutual information), e.g., InfoVAE [9,10], or which separate important factors of variations in the data (disentangling), e.g., [18,20,21]. Although these methods report good results on occasions, there seem to be little evidence that breaking the variational bound brings systematical improvements [12,15].

For our model, the analysis presented in Section 4.3.1 suggests that our objective (which is a proper lower bound on the likelihood) encourages the encoding of the major source of variation, that of the originating mixture component, through the categorical variable without any extra alterations. At the same time, it should be noted that our goal is not the interpretability of the learned representations or their reuse outside the VAEs models. Our focus is on generations reflecting the underlying multi-modal distribution over the original data space.

Second, the simplifying conditional independence assumptions for the data dimensions factored into the simple Gaussian decoder $p_{\theta }\left(x|z\right)$ have been challenged in the context of modelling data with strong internal dependencies. More powerful decoders with autoregressive architectures have been proposed for modelling images, e.g., PixelVAE [22], or sequentially dependent data such as speech and sound, e.g., VRNN [23]. In our model, we use a hierarchical decoder $p_{\theta }\left(x|z,c\right)$ corresponding to the cluster-like structure we assume for the data space. However, in this work, we stick to the simple independence assumption for the data dimensions. Augmenting our method with stronger decoder should, in principle, be possible and it is open for future investigation.

Third, the insufficient flexibility of the variational posterior $q_{\phi }\left(z|x\right)$ to approximate the true posterior $p_{\theta }\left(z|x\right)$ has led to proposals for more expressive posterior classes. For example, a rather successful approach is based on chaining invertible transformations of the latent variable [3,5]. While increasing the flexibility of the approximate posterior improves the modelling objective through better reconstructions, without accompanied enhancements of the prior it does not guarantee better generations.

This has been recognised and addressed by the fourth group of improvements that focuses on the model prior and that our method pursues. These build on the observation that overly-simple priors can be source of excessive regularization, limiting the success of the VAE models [6,19]. For example, the authors in [11,24] replace the distributional class of the prior (together with the posterior) by von Mises–Fisher distributions with potentially better characteristics for high-dimensional data with hyperspherical latent space.

More related to ours are methods that suggest to learn the prior. The VLVAE [6] uses the autoregressive flows in the prior that are equivalent to the inverse autoregressive flows in the posterior [5]. The increased richness of the encoding and prior distributions leads to higher quality generations; however, the prior cannot be used to generate from selected parts of the data space, as our model can.

The VampPrior [17] proposes constructing the prior as a mixture of the variational posteriors over a learned set of pseudo-inputs. These could be interpreted as learned cluster prototypes of the data. However, the model does not learn the importance of the components in the mixture, and it does not align the prior and posteriors at an individual component level as our model does. Instead, it pushes the posteriors to align with the overall prior mixture that diminishes the models ability to correctly generate from the individual components of multimodal data. In [25], they use the aggregated posterior as the prior by directly estimating the KL divergence without modeling the aggregated posterior explicitly, while using a kernel density trick. However, because their prior is implicit, they cannot sample from the prior directly. Instead, they sample from the aggregated posterior. Moreover, the model simillarly to VampPrior does not learn the importance of the components in the mixture.

The continuous-discrete decomposition of the latent space similar to ours have been used for data clustering through generative model presented in [7,26]. The first combines the VAE with a Gaussian mixture model through two stage procedure mimicking the independence assumptions in their inference model. The latter assumes (conditional) independence in the generative and inference models and extends to a full Bayesian formulation through the use of hyper-priors. Their complex model formulation exhibits some over-regularization issues that, to the authors acknowledge, are challenging to control.

Options for freeing the distributional class of the latent representations through Bayesian non-parametrics have been explored, for example, in [8,27,28]. The learned structures in the latent representations greatly increase the generative capabilities, including also the (hierarchical) clustering ability. However, this comes at a cost of complex models that are tricky to train in a stable manner. In contrast, our model is elegantly simple and easy to train.

## 4. VAE with Data-Dependent Conditional Priors

The mathematically and practically convenient assumption of the factorial Gaussian approximate posterior $q_{\phi }\left(z|x\right)$ has been previously contested as one of the major limitations of the basic VAE architecture. For complex data distributions p(x), the simple Gaussian $q_{\phi }\left(z|x\right)$ may not be flexible enough to approximate well the true posterior $p_{\theta }\left(z|x\right)$.

Even though various methods have been proposed for enriching the posterior distributions, as we mention in Section 3, by learning latent representations more appropriate for the complex data structures they cannot guarantee better generations. In order to achieve this a closer match between the posterior and prior distributions used for sampling the latent variables during inference and data generations, respectively, is required.

We propose a new VAE formulation, conditional prior VAE (CP-VAE), with a conditionally structured latent representation that encourages a better match between the prior and the posterior distributions by jointly learning their parameters from the data.

### 4.1. Two-Level Generative Process

We consider a two-level hierarchical generative process for the observed data where two latent variables c and z are introduced in addition to the observed variables x. Variable c is a K−way categorical latent variable, and z is a D−dimensional continuous latent variable. To generate x, we first sample c sample from its prior, p(c), and then a continuous latent variable z is sampled from the learned conditional distribution $p_{\varphi }\left(z|c\right)$. Finally, a sample is drawn from $p_{\theta }\left(x|z,c\right)$, parameterized by the decoder network. The joint probability can be written as:

(8) $$p\left(x,z,c\right)=p_{\theta }\left(x|z,c\right)p_{\varphi }\left(z,c\right)$$

where, the joint prior distribution is equal to $p_{\varphi }\left(z,c\right)=p_{\varphi }\left(z|c\right)p\left(c\right)$.

We assume a uniform categorical as a prior distribution for the discrete component c, so that, for each of the *K* categories $p(c_{k})=1/K,\;k=1,\ldots ,K$, which encourages every component to be used. The conditionals of the continuous component are factorised Gaussians with learnable means and variances.

(9) $$p_{\varphi }\left(z|c_{k}\right)=\prod_{i}p_{\varphi }\left(z_{i}|c_{k}\right)=\prod_{i}N\left(z_{i}\;\left|\;\mu_{ik},\sigma_{ik}^{2}\right)\right),\;k=1,\ldots ,K\;.$$

The compositional prior we propose is well suited for generations of new samples from multimodal data distributions mixing multiple distributional components. In contrast to sampling from a simple isotropic Gaussian prior that concentrates symmetrically around the origin, we can sample the latent code from discontinuous parts of the latent space. These are expected to represent data clusters corresponding to the originating distributional mixing.

In addition, the variations encoded into the continuous part of the latent space are also sampled conditionally and therefore are better adapted to represent the important factors of data variations within the distributional clusters. This is in contrast to the single common continuous distribution of the basic VAE (Section 2.2) or VAEs with similar continuous-discrete composition of the latents as ours, which, however, assume independence between the two parts of the latent representation [21], which we discuss in detail in Section 5.1.

The data conditional $p_{\theta }\left(x|z,c\right)$ is parametrised by a decoder network $d_{\theta }\left(z,c\right)$ as a Bernoulli $\left(x\;|\;d_{\theta }\left(z,c\right)\right)$ or a Gaussian $N\left(x\;|\;d_{\theta }\left(z,c\right),\sigma^{2}I\right)$ distribution, depending on the nature of the data x.

#### Data-Dependent Conditional Priors

There is no straightforward way to fix the parameters φ=(μ,σ) in the distributions Equation (9) for each of the conditioning categories $c_{k}$ a priori. Instead of placing hyper-priors on the parameters and expanding to full hierarchical Bayesian modelling, we estimate the prior parameters from the data through a relatively simple procedure that resembles the empirical Bayes technique [29].

**Figure 1:**
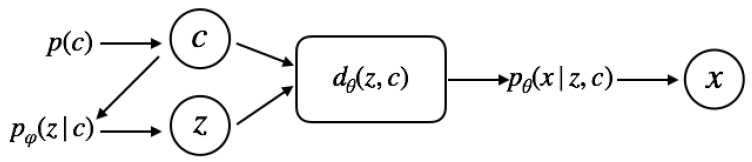
To generate new examples from the learned data distribution $p_{\theta }\left(x\right)$, we sample the discrete and continuous latent variables from the two-level prior and pass those through the decoder.

As explained in Section 4.3, the conditional $p_{\varphi }\left(z|c\right)$ enters our objective function through a KL divergence term. Therefore, the prior parameters φ can be optimized by backpropagation together with learning the encoder and decoder parameters ϕ and θ. Once the model is trained, all of the parameters are fixed and the learned prior $p_{\varphi }\left(z|c\right)$ can be used in the ancestral sampling procedure that is described above to generate new data samples, Figure 1.

### 4.2. Inference Model

As in standard VAEs, we employ amortized variational inference to learn the unknown data distribution. We use the approximate posterior distribution

(10) $$q_{\phi }\left(z,c|x\right)=q_{\phi }\left(z|x,c\right)q_{\phi }\left(c|x\right)$$

in place of the intractable posterior $p_{\theta }\left(z,c|x\right)$.

Our approximate posterior replicates the two-level hierarchical structure of the prior. In this way, we ensure that the latent samples are structurally equivalent both during inference and new samples generations. This is not the case in other hierarchical latent models that rely on simplifying mean field assumptions for the posterior inference [7,26].

**Figure 2:**
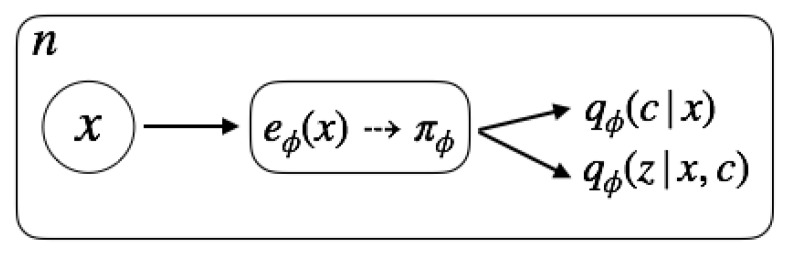
The encoder infers the parameters of the discrete and continuous approximate posteriors using a gated layer for the hierarchical conditioning. First, it outputs the parameters of the discrete latent variable, $\pi_{\phi }$. Subsequently, there is an extra layer that takes as input $\pi_{\phi }$ concatenated with the last hidden layer of the encoder and infers the parameters of the continuous latent variable.

We use encoder network with a gated layer $e_{\phi }\left(x\right)=\left(\pi_{\phi }\left(x\right),\mu_{\phi }\left(x,\pi \right),\sigma_{\phi }\left(x,\pi \right)\right)$ for the amortized inference of the variational approximate posteriors, Figure 2

$$\begin{array}{c} q_{\phi }\left(c|x\right)=\mathrm{Cat}\left(\pi_{\phi }\left(x\right)\right)\\ q_{\phi }\left(z|x,c\right)=N\left(\mu_{\phi }\left(x,\pi \right),\mathrm{diag}\left(\sigma_{\phi }^{2}\left(x,\pi \right)\right)\right)\;. \end{array}$$

### 4.3. Optimization Objective

As customary in variational inference methods, our optimization objective is the maximization of the lower bound on the data log-likelihood

(11) $$\begin{array}{c} L_{\theta ,\phi }\left(x\right)=\underset{A}{\underset{︸}{E_{q_{\phi }\left(z,c|x\right)}\log p_{\theta }\left(x|z,c\right)}}-\underset{B}{\underset{︸}{\mathrm{KL}\left(q_{\phi }\left(z,c|x\right)\left|\right|p_{\varphi }\left(z,c\right)\right)}}\;. \end{array}$$

This is a straightforward adaptation of the bound from Equation (6) to the compositional latent code (z,c) with similar interpretations for the *A* and *B* terms. Using the prior and posterior distribution decompositions from Equation (10) the KL term in *B* can be rewritten as a sum of two KL divergences that are more amenable to practical implementation: B1 for the continuous conditional distributions and B2 for the discrete.

(12) $$\begin{array}{cc} \underset{B}{\underset{︸}{\mathrm{KL}\left(q_{\phi }\left(z,c|x\right)∥p_{\varphi }\left(z,c\right)\right)}} & =\underset{B1}{\underset{︸}{E_{q_{\phi }\left(c|x\right)}\mathrm{KL}\left(q_{\phi }\left(z|x,c\right)\left|\right|p_{\varphi }\left(z|c\right)\right)}}+\underset{B2}{\underset{︸}{\mathrm{KL}\left(q_{\phi }\left(c|x\right)\left|\right|p\left(c\right)\right)}} \end{array}$$

The first term B1 can be seen as a weighted average of the KL divergences between the posterior and prior conditionals. The weights are the probabilities of the posterior categorical distribution, so that the two conditionals are pushed together more strongly for those observations x and latent categories $c_{k}$ to which the model assigns high probability. The KLs can be conveniently evaluated in a closed form as both the posterior and the prior conditionals are diagonal Gaussians.

The minimization of the KL divergence between the categorical posterior and the fixed uniform prior in the second term B2 is equivalent to maximizing the entropy of the categorical posterior $H\left(q_{\phi }\left(c|x\right)\right)$ (up to a constant).

(13) $$\underset{B2}{\underset{︸}{\mathrm{KL}\left(q_{\phi }\left(c|x\right)\left|\right|p\left(c\right)\right)}}=-H\left(q_{\phi }\left(c|x\right)\right)+\log K$$

We train the model by a stochastic gradient-based algorithm (Adam [30]). As the gradients of the variational lower bound $L_{\theta ,\phi }$ with respect to the model parameters are intractable, we use the usual well-established Monte-Carlo methods for their estimation.

For the decoder parameters θ, the gradient is estimated as the sample gradient of the conditional log-likelihood with the latent z and c sampled from the approximate posterior.

(14) $$\begin{array}{c} \nabla_{\theta }L_{\theta ,\phi }\left(x\right)\approx \nabla_{\theta }\log p_{\theta }\left(x|z,c\right),\;\left(z,c\right)∼q_{\phi }\left(z,c|x\right) \end{array}$$

For the encoder parameters ϕ, we use the pathwise gradient estimators [31] based on the standard location-scale $z=f_{\phi }\left(\tilde{z}\right)$ and Gumbel–Softmax [32]$c=g_{\phi }\left(\tilde{c}\right)$ reparametrizations with the auxiliary $\tilde{z}∼N\left(0,1\right)$ sampled from the standard normal and $\tilde{c}$ sampled from the Gumbel(0,1) distribution.

(15) $$\begin{array}{cc} \nabla_{\phi }L_{\theta ,\phi }\left(x\right)\approx & \nabla_{\phi }\log p_{\theta }\left(x,f_{\phi }\left(\tilde{z}\right),g_{\phi }\left(\tilde{c}\right)\right)-\nabla_{\phi }\log q_{\phi }\left(f_{\phi }\left(\tilde{z}\right),g_{\phi }\left(\tilde{c}\right)|x\right),\;\tilde{z}∼N\left(0,1\right),\;\tilde{c}∼\mathrm{Gumbel}\left(0,1\right) \end{array}$$

Finally, the gradients with respect to the parameters φ of the conditional prior are estimated alongside the gradients of the decoder under the same sampling of the latents.

(16) $$\begin{array}{c} \nabla_{\varphi }L_{\theta ,\phi }\left(x\right)\approx -\nabla_{\varphi }\log p_{\varphi }\left(z|c\right),\;\left(z,c\right)∼q_{\phi }\left(z,c|x\right) \end{array}$$

#### 4.3.1. Analysis of the Objective

The KL divergence in term *B* of the objective Equation (11) has important regularization effects on the model learning. We expand on the discussion of these in the standard VAE objective Equation (6) from [9] to analyse our more complex model formulation.

There are two major issues that optimizing the reconstruction term *A* of the objective Equation (11) in isolation could cause. First, the model could completely ignore the categorical component of the latent representation c by encoding all of the data points x into a single category with a probability $q_{\phi }\left(c_{k}|x\right)=1$ for all x. All of the variation in the data x would then be captured within the continuous component of the latent representation through the single continuous posterior $q_{\phi }\left(z|x,c_{k}\right)$. While this would not diminish the ability of the model to reconstruct the observed data and, therefore, would not decrease the reconstruction part of the objective *A*, it would degrade the generative properties of our model. Specifically, with all of the data clusters pushed into a single categorical component and distributed within the continuous latent space, we would have no leverage for generating samples from the individual data distributional components, which is one of the major requirements for our method. This pathological case is essentially equivalent to learning with the standard VAE.

Second, maximizing the log-likelihood in *A* naturally pushes the continuous posteriors to be concentrated around their means in disjoint parts of the continuous latent space with variances tending to zero, as discussed in [9]. For such posteriors, the model could learn very specific decoding, yielding very good reconstructions with very high log-likelihoods $p_{\theta }\left(x|z,c\right)$. However, the generations would again suffer as the prior used for the ancestral sampling would not cover the same areas of the latent space as used during the inference.

To analyse the reguralization effect of term *B* in the objective Equation (11) on the learning, we decompose the expected KL divergence into three terms and a constant (see proof in Appendix A.1):

(17) $$\begin{array}{ll} E_{p(x)}\mathrm{KL}\left(q_{\phi }\left(z,c|x\right)∥p_{\varphi }\left(z,c\right)\right)\; & =I_{q}\left((z,c),x\right)\;\\ & +E_{q_{\phi }\left(c\right)}\mathrm{KL}\left(q_{\phi }\left(z|c\right)∥p_{\varphi }\left(z|c\right)\right)\;\\ & -H\left(q_{\phi }\left(c\right)\right)+\log K\;. \end{array}$$

The first is the mutual information of the composite latent variable (z,c) and the data x under the posterior distribution *q*. Minimization of the KL divergence in Equation (11) pushes the mutual information between the two to be low and, therefore, prevents the overfitting of the latent representation to the training data described in the second point above.

The third term is the negative entropy of the marginal categorical posterior whose empirical evaluation over the data sample $S={\left\{x_{i}\right\}}_{i=1}^{n}$ is often referred to as the aggregated posterior [17,33].

(18) $$q_{\phi }\left(c\right)=E_{p(x)}q_{\phi }\left(c|x\right)\approx \frac{1}{N}\sum_{i}^{N}q_{\phi }\left(c|x_{i}\right)$$

The regularizer maximizes the entropy of this distribution, thus encouraging the model to use evenly all of the categories of the discrete latent code counteracting the pathological case of the first point above.

Finally, the middle term pushes the marginalized conditional posteriors of the continuous latent variable z to be close to the priors conditioned on the corresponding categories. It helps to distribute the variations in the data into the continuous component of the latent space in agreement between the inferential posteriors and the learned generative priors. It does so for each latent category $c_{k}$ separately, putting more or less weights on the alignment, as per the importance of the latent categories established through the categorical marginal posterior $q_{\phi }\left(c\right)$. It is this term in the objective of our VAE formulation that safeguards the generative properties of the model by matching the inferential posteriors and the learned generative priors used in the ancestral sampling procedure for new data examples.

## 5. VAE with Continuous and Discrete Components

We unify and analyse the objective under different assumptions for the joint distribution of continuous and discrete latent variables $p_{\phi }\left(z,c\right)$ and $q_{\phi }\left(z,c|x\right)$ in order to justify our decisions for the inference and generative model.

As in the vanila VAE, the different variations of VAE with continuous and discrete latent variables jointly optimize the generative and the inference model. Using discrete latent variables we impose a categorical distribution as the output of the encoder. We first perform a decomposition of the objective given by Equation (11) and then apply different independence assumptions about the inference and generative models.

(19) $$\begin{array}{cc} L(\theta ,\phi )\; & =\underset{A}{\underset{︸}{E_{q_{\phi }\left(z,c,x\right)}\left[\log p_{\theta }\left(x|z,c\right)\right]}}-\underset{B}{\underset{︸}{I_{q}\left((z,c),x)\right)}}-\underset{C}{\underset{︸}{\mathrm{KL}(q_{\phi }\left(z,c\right)∥p_{\varphi }\left(z,c\right))}} \end{array}$$

### 5.1. Comparing the Alternative Models

To better understand the various modifications of the VAE objective with continuous and categorical latent variables, we review the possible independence assumptions for the inference and generative models as summarized in Table 1. For the marginal posterior we assume the same decomposition as for the corresponding prior, i.e., $p_{\varphi }\left(z|c\right)p\left(c\right)=q_{\phi }\left(z|c\right)q_{\phi }\left(c\right)\;\mathrm{and}\;p\left(z\right)p\left(c\right)=q_{\phi }\left(z\right)q_{\phi }\left(c\right)$.

As we show in Appendix A.2, Equation (19) can be rewritten in the general form of Equation (20) for all the models considered in Table 1.

(20) $$\begin{array}{cc} L(\phi ,\theta ) & =\underset{A}{\underset{︸}{E_{q_{\phi }\left(z,c,x\right)}\left[\log p_{\theta }\left(x|z,c\right)\right]}}-B1-\underset{B2}{\underset{︸}{I_{q}\left(c,x\right)}}-C1-\underset{C2}{\underset{︸}{\mathrm{KL}(q_{\phi }\left(c\right)∥p\left(c\right))}} \end{array}$$

The terms A,B2 and C2 remain the same in all of the models, terms B1 and C1 way, as per the independence assumptions listed in Table 1. *A* is the negative reconstruction cost. Term B2, is the mutual information in the inference model between the discrete latent variable and the observed data. Through minimizing this mutual information we encourage x to be independent from the discrete latent variable. Term C2 matches the discrete marginal posterior $q_{\phi }\left(c\right)$ to the prior p(c).

CP-VAE

In the proposed model where we do not make any independence assumption about the approximate posterior, $q_{\phi }\left(z,c|x\right)=q_{\phi }\left(z|x,c\right)q_{\phi }\left(c|x\right)$ and the prior, $p_{\varphi }\left(z|c\right)=p_{\varphi }\left(z|c\right)p\left(c\right)$.

The term B1 is the mutual information between the continuous latent variable z given the discrete latent variable c and the data x given the discrete latent variable c. Inferring the continuous latent variable z from x and c could result in only using the information from x ignoring the discrete latent variable c. By minimizing B1 term, we encourage z|c and x|c to be decoupled by removing the information of the data distribution given a category from the continuous latent variables. In this way, we ensure that, when inferring the continuous latent variable z, the discrete latent variable will be used. Moreover, minimizing this term penalizes the first term, the negative reconstruction error, helping to avoid over-fitting.

The term C1 matches the marginalized conditional posteriors of the continuous latent variable z, $q_{\phi }\left(z|c\right)$ to the priors conditioned on the corresponding categories, $p_{\varphi }\left(z|c\right)$ (see also Section 4.3.1).

INDq model

In INDq model, we assume conditional independence between the continuous and discrete latent variables, $q_{\phi }\left(z,c|x\right)=q_{\phi }\left(z|x\right)q_{\phi }\left(c|x\right)$ without making any independence assumption about the prior, $p_{\varphi }\left(z,c\right)=p_{\varphi }\left(z|c\right)p\left(c\right)$. The continuous latent variable z is inferred from the observed data, while, in our model, it is inferred from the observed data and the discrete latent variable c.

B1 term encourages the approximate continuous posterior, $q_{\phi }\left(z|x\right)$, to be close to the conditional distribution of the continuous latent variable z given the discrete latent variable c, $q_{\phi }\left(z|c\right)$. This means that, even if the discrete latent variable c is not used to infer the continuous z, the continuous latent variable is encouraged to contain information for the corresponding category, but it is not ensured that it will used it like in our model. The term C1 is the same as in the CP-VAE with the same effect.

These assumptions are made by the Variational Deep Embedding (VaDE) paper [7], where the authors proposed a clustering framework.

INDp model

In INDp model, we do not make any independence assumption about the approximate posterior $q_{\phi }\left(z,c|x\right)=q_{\phi }\left(z|x,c\right)q_{\phi }\left(c|x\right)$, but we assume marginal independent priors $p_{\varphi }\left(z,c\right)=p\left(z\right)p\left(c\right)$.

In this model, similarly to our model, the continuous latent variable z is inferred from the observed data and the discrete latent variable c.

B1 term is the mutual information between the continuous latent variable z and (c,x) pair governed by the joint distribution $q_{\phi }\left(c,x\right)$. Minimizing this mutual information, we encourage z and (c,x) to become independent, discouraging z to contain any information about the discrete latent variable c and the data x, even though the discrete latent variable c is used to infer the continuous z.

C1 is the KL divergence between the marginalized continuous posterior $q_{\phi }\left(z\right)$ and the prior p(z). This helps to produce realistic samples without relying on any information regarding the corresponding category.

In this model, none of the terms ensure that the discrete latent variable c will not be ignored while inferring the continuous latent variable z. This, in combination with the non-appearance of the discrete latent variable c in the KL term C1, makes it infeasible to generate samples from a specific category, in contrast to our proposed model.

The INDp assumptions are used in the semi-supervised model by Kingma et al. in [34], where the discrete label is treated as a latent variable when missing. Their model is augmented with a discriminative loss in order to learn better the categorical approximate posterior while using the labelled data. Without the use of supervision, there is no guarantee that it would be able to generate samples from specific categories. Gaussian Mixture Variational Autoencoder (GMVAE) [26] is built upon the semi-supervised model [34] adding an extra latent variable.

INDqp model

INDqp model assumes conditional independence between the continuous and discrete latent variables and marginal independent priors. In this case, the continuous latent variable z is only inferred from the observed data, the same as in the INDq model.

B1 term minimizes the mutual information between the continuous latent variable z and x. Encouraging z and x to become independent, we help to avoid over-fitting by preventing the learning of a unique z for each x (also see Section 4.3.1). The C1 term is the same as in the INDp model. It matches the marginalized continuous posterior $q_{\phi }\left(z\right)$ to the prior p(z).

In contrast to our proposed model, in INDqp, none of the terms in the objective prevent the model from ignoring the discrete latent variables or guarantees samples from a specific category.

This was also experimentally found in [21], where the same independence assumptions are used in order to learn disentangled representations in an unsupervised manner. To overcome this issue, they added weights to control the capacities of the discrete and continuous latent variables. These weights are modified separately during the training (like an annealing procedure) forcing the model to encode information both in the discrete and continuous variables. Moreover, the same model is also used under the setting of continual learning [13], where a mutual information regularizer is added in order to overcome this issue.

### 5.2. Assuming Uniform Approximate Categorical Posterior

In this section, we examine the special case where instead of inferring the categorical posterior as in the models above, we assume that it follows a uniform distribution over *K* components $q_{\phi }\left(c|x\right)∼\frac{1}{K}$. We show that the vanilla VAE is a special case of the INDqp model.

Assuming that the categorical posterior follows the uniform distribution, the marginal categorical posterior $q_{\phi }\left(c\right)$ is equal to $\frac{1}{K}$, $\left(q_{\phi }\left(c\right)=\sum_{n}^{N}q_{\phi }\left(c,x\right)=\sum_{n}q_{\phi }\left(c|x\right)p\left(x\right)=\frac{1}{K}\sum_{n}p\left(x\right)=\frac{1}{K}\times 1=\frac{1}{K}\right)$ and the terms B2 and C2 in Equation (20) are equal to zero $\left(I_{q}\left(c,x\right)=0\;\mathrm{and}\mathrm{KL}\left(q_{\phi }\left(c\right)∥p\left(c\right)\right)=0\right)$. The objective of the INDqp model becomes

(21) $$\begin{array}{cc} L(\phi ,\theta ) & =\underset{A}{\underset{︸}{E_{q_{\phi }\left(z,x\right)}\left[\log p_{\theta }\left(x|z,c\right)\right]}}-\underset{B1}{\underset{︸}{I_{q}\left(z,x\right)}}-\underset{C1}{\underset{︸}{\mathrm{KL}(q\left(z\right)∥p_{\theta }\left(z\right))}}\\ \; & =E_{p(x)}\left[E_{q_{\phi }\left(z|x\right)}\left[\log p_{\theta }\left(x|z,c\right)\right]-\mathrm{KL}(q\left(z|x\right)∥p_{\theta }\left(z\right)\right] \end{array}$$

which is the VAE objective with an extra c in the *A* term. Given that we do not infer the categorical posterior, this extra c does not influence the model.

## 6. Empirical Evaluation

We validate our new conditional prior (CP-VAE) model through experiments (the implementation of our method together with the settings for replication of our experiments is available from our Bitbucket repository https://bitbucket.org/dmmlgeneva/cp-vae/) over synthetic data and three image datasets (MNIST [35], FashionMNIST [36] and Omniglot [37]). We compare the results with those produced by standard VAE (VAE), VAE with Mixture of Gaussian prior (MoG), and VAE with VampPrior (VP) [17], and the three combinations of discrete and continuous latent variable models discussed in Section 4.

We use the same structure of the encoder and decoder networks for all the methods in all our experiments not to obfuscate the analysis of the benefits of our method by various tweaks in the model architecture.

We set the dimensions of the continuous latent variable to 40, we use simple feed-forward networks with two hidden layers of 300 units each for both the encoder and the decoder, we initialise the weights according to Glorots method [38], and we utilize the gating mechanism of [39] as the element-wise non-linearity.

We trained all of the models while using ADAM optimizer [30] with learning rate $5\times 10^{-4}$ and early stopping based on the stability of the objective over a validation-set. We use a linear annealing/warm-up scheme of 100 epochs to avoid pathological local minima and numerical issues during training [16], during which the KL regularization in the objective is annealed from 0 to 1 during training.

For generating new data examples, we use the ancestral sampling strategy with the latent variables being sampled from the respective prior distributions of each method. In the simple VAE, this is from the standard normal Gaussian z∼p(z)=N(z|0,I). In the MoG model it is from the set of learned Gaussian components $z∼p_{\varphi }\left(z\right)=\frac{1}{K}\sum_{i}^{K}N\left(z|\mu_{k},\mathrm{diag}\left(\sigma_{k}\right)\right)$ with equal weighting. For VP, it is from the mixture of variational posteriors $z∼p_{\varphi }\left(z\right)=\frac{1}{K}\sum_{i}^{K}q_{\phi }\left(z|u_{k}\right)$ over the learned set of pseudo-inputs $U={\left\{u_{k}\right\}}_{i=1}^{K}$, which first have to be passed through the encoder network. For INDqp and INDp, we sample the continuous latent component from the standard normal Gaussian z∼p(z)=N(z|0,I) and from the empirical aggregated posterior Equation (18) for the discrete component $c∼q_{\phi }\left(c\right)$ For our method, we follow the two level-generative process described in Section 4.1, where we use the learned conditional priors for each of the categories for sampling the continuous latent component $z∼p_{\varphi }\left(z|c=c_{k}\right)=N\left(z|\mu_{k},\mathrm{diag}\left(\sigma_{k}\right)\right)$ and the empirical aggregated posterior Equation (18) for the discrete component $c∼q_{\phi }\left(c\right)$. We follow a similar procedure for the INDq model.

### 6.1. Synthetic Data Experiments

In this section, we demonstrate the effectiveness of the CP-VAE method through experiments over synthetic data. We use a toy dataset with 50,000 examples x∈R generated from a Gaussian mixture with two equally weighted components $x∼p\left(x\right)=\frac{1}{2}\left(N(0.3,0.05)+N(0.7,0.05)\right)$.

This simple set-up allows us to better understand the strengths and weaknesses of the method in terms of its density estimation performance for a known and rather simple ground-truth data distribution.

We use two experimental set-ups because, in real-life problems, the number of distributional clusters in the data (the number of mixture components) may not be known or even easy to estimate:

- **known** number of components: discrete latent variable c with two categories (corresponding to the ground-truth two mixture components)
- **unknown** number of components: discrete latent variable c with 150 categories

**Figure 3:**
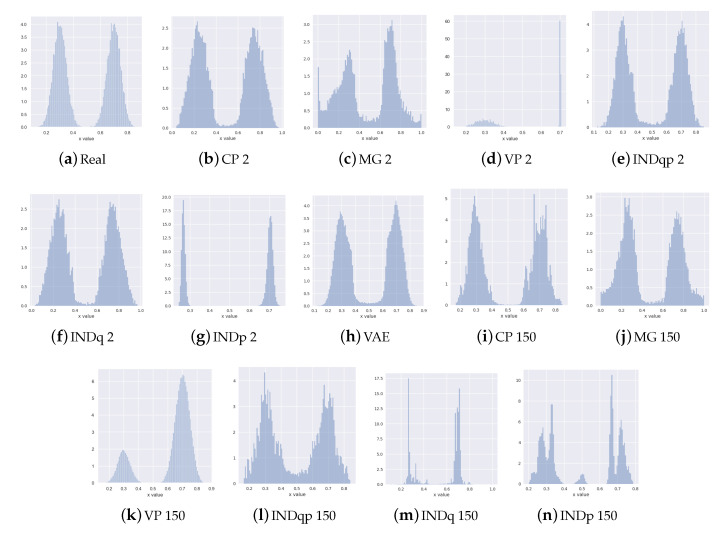
Histograms of the data generated from the ground-truth $x∼p\left(x\right)=\frac{1}{2}\left(N(0.3,0.05)+N(0.7,0.05)\right)$ and the learned distributions using CP-VAE, MoG, VampPrior INDq, INDp, INDqp, with 2 and 150 categories and VAE. Our CP method can recover the bi-modal structure of the data correctly, irrespective of the number of categories used for the latent categorical component.

In Figure 3, we present histograms of data generated from the ground truth and the learned distributions. As we can see, our method (CP) correctly recovers the bi-modal structure of the data for both set-ups. This is important for practical utility of the method in situations where the domain knowledge does not provide an indication on the number of underlying generative clusters. With high enough number of categories within the discrete latent, our method can recover the correct multi-modal structure of the data. INDq has similar behaviour to our model when we use a discrete latent variable c with two categories, which confirms the importance of learning the conditional prior $p_{\varphi }\left(z|c\right)$ instead of assuming marginal independent prior. When the number of categories is 150, it has difficulties to recover the structure of the data in contrast to our model.

**Figure 4:**
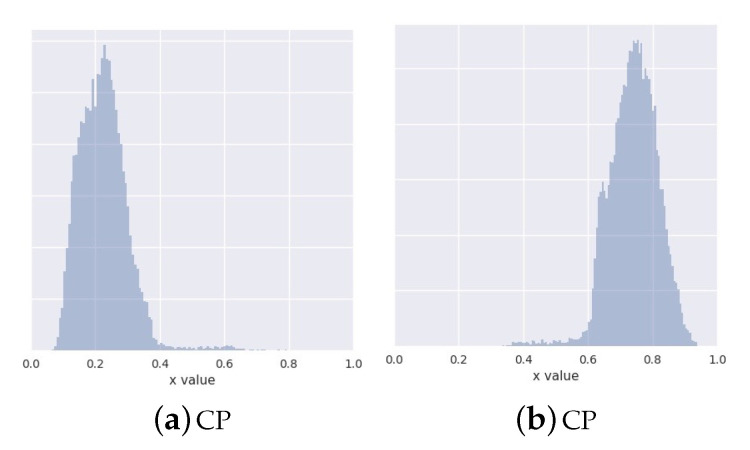
Histograms of conditionally generate samples using our conditional prior variational autoencoder (CP-VAE) model with latent discrete variable with two categories. In subfigure **a** we conditionally generate samples from the first category and in subfigure **b** we conditionally generate samples from the first category.

Because of the simplicity of this set-up, even methods that do not adjust their priors to the disjoint learned representation, such as the simple VAE is able to recover the multimodal structure of the data at generation time. However, VAE in contrast to CP-VAE, Figure 4, because of the nature of the model, is not able to conditionally generate samples. MoG and VP have difficulties to recover the structure of the data when a small number of components/pseudo-inputs is used. This seems to improve when the number of components/pseudo-inputs is increased to 150. In contrast, INDqp and INDp have difficulties to recover the structure of the data when a large number of components is used, but this is improved when the exact number of components is used. This can be problematic in practice when the number of mixture components is not known or difficult to estimate.

We further explore how our model handles the excess capacity within the categorical latent variable. For this, we focus on the 150-category case and generate data by sampling the discrete latent variable (a) from the marginal posterior $c∼q_{\phi }\left(c\right)$, (b) from the uniform prior $c∼p\left(c\right)=\frac{1}{K}$.

**Figure 5:**
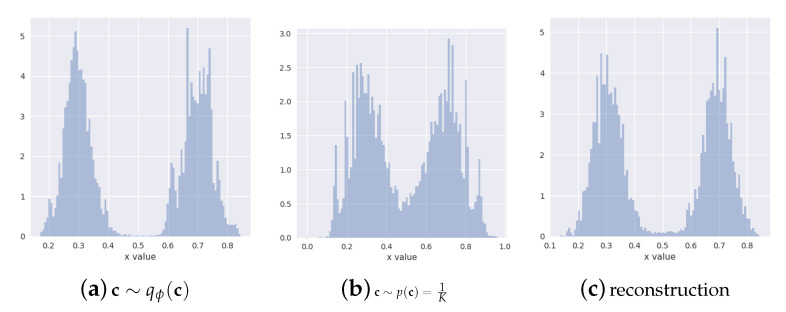
CP-VAE (150-category case) generations sampled from the marginal posterior (**a**) and from the uniform prior (**b**) and CP-VAE reconstructions (**c**). The CP-VAE learns to ignore the excess capacity of the disjoint latent space by assigning near-zero probability to some of the categories in the discrete latent space. These parts of the latent space are ignored for the reconstructions and by sampling the categorical variable from the marginal posterior $q_{\phi }\left(c\right)$ can correctly be ignored also for the generations.

When comparing the two in Figure 5, we see that, unlike the generations sampled from the marginal posterior, the generations from the uniform prior display some mixing artifacts. This suggests that our model learns to ignore the excess capacity by assigning low marginal probabilities $q_{\phi }\left(c_{k}\right)\approx 0$ to some of the categories. The continuous latent representations that correspond to these parts of the disjoint latent space are irrelevant for both the reconstructions and the generations due to our weighted KL formulation in B1 of Equation (12).

### 6.2. Real Data Experiments

For the real-data experiments, we use three image datasets, MNIST [35], FashionMNIST [36] and Omniglot [37], commonly used for the evaluation of generative models. We use the dynamically binarized versions of the datasets, as in [40], with the following train-validation-test splits: for MNIST and FashionMNIST 50,000–10,000–10,000, for Omniglot 23,000–1345-8070.

We examine the ability of our model to generate new examples from the underlying distributional clusters. For this, we trained our model (CP-VAE) over the FashionMNIST data with 150 categories in the discrete latent variable and compared it to the INDq, INDp, and INDqp models.

**Figure 6:**
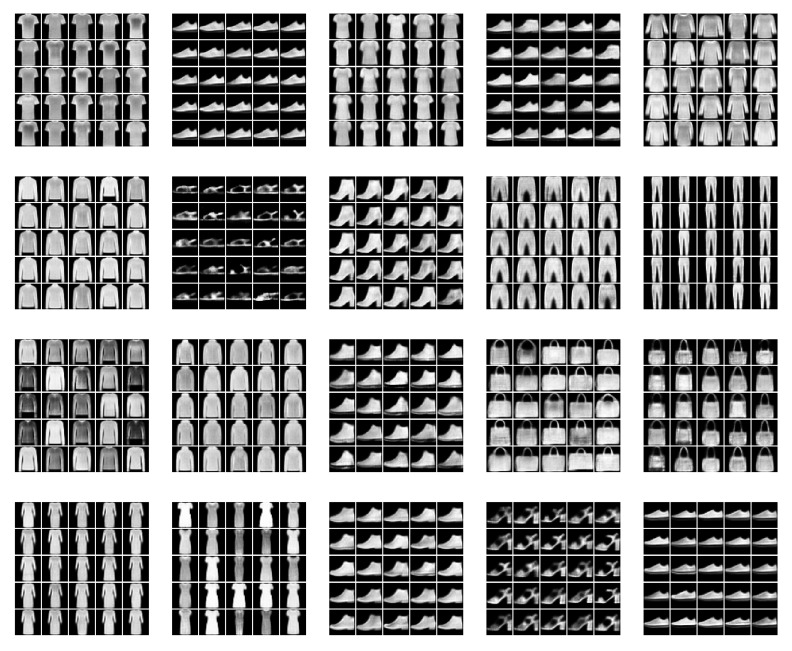
New data examples from the FashionMNIST generated by our CP-VAE model with latent discrete variable with 150 categories. Examples in the same subplot were generated from the same discrete category. To generate the samples we randomly use 20 categories with probability higher than 1/150. CP-VAE accurately captures not only the main source of variation of the data, but can also find subcategories among each main category, in a totally unsupervised manner. For example we condition on category 61 and we can see in the 2nd subplot of the second row that it generates flat sandals while when we condition on category 34 in the fourth subplot of the fourth row it generates sandals with heels.

**Figure 7:**
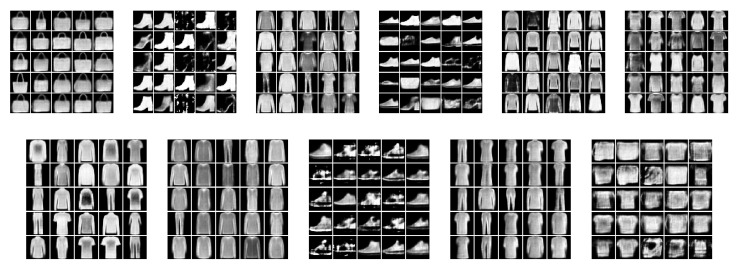
New data examples from the FashionMNIST generated by INDq model with a latent discrete variable with 150 categories. Examples in the same subplot were generated from the same discrete category. To generate the samples, we use all of the categories with probability higher than 1/150. INDq model is not always able to conditionally generate new samples from the individual modes of the underlying distribution but generates a mix of images from different modes in some subplots. For example in the 1st subplot of the second row it mixes t-shirts, dresses, pullovers, and trousers, and in the third subplot of the second row it mixes flat sandals with sneakers and ankle boots.

**Figure 8:**
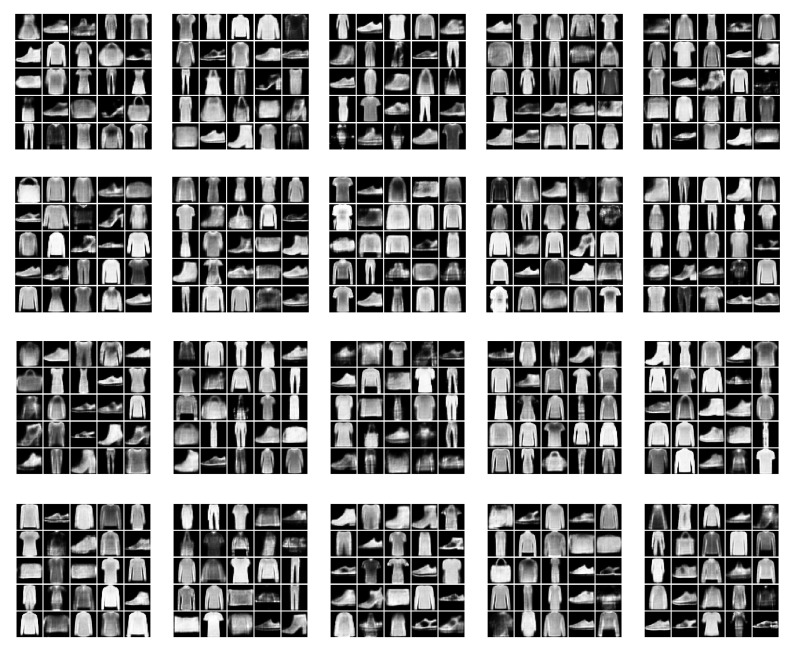
New data examples from the FashionMNIST generated by INDp model (1st–2nd row) and INDqp model (3rd–4th row) with a latent discrete variable with 150 categories. Samples in the same subplot were generated from the same discrete category. For both models we randomly pick 10 categories with probability higher than 1/150 in order to generate the samples. INDp and INDqp models are both not able to conditionally generate new samples from the individual modes of the underlying distribution, but generate a mix of images from different modes.

Figure 6 illustrates FashionMNIST generations using our model, Figure 7 generations using INDq model and Figure 8 sample generations using INDp model (1st row) and INDqp model (second row). For all the models the examples in each of the subplots were generated by fixing the discrete latent variable to one category and sampling the continuous latent from the corresponding learned prior for the CP-VAE and INDq models and the standard normal distribution for the INDp and INDqp. Moreover, in all the cases, we only consider the categories of the discrete latent variable with probability higher than 1/150 (this is the probability assuming the categorical marginal posterior follows the uniform distribution).

**Figure 9:**
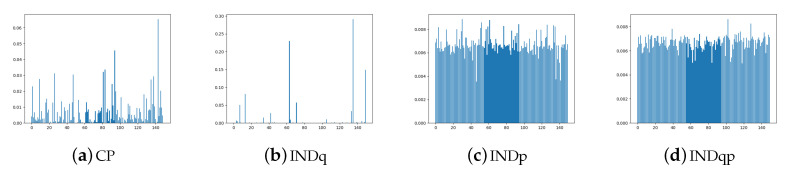
FashionMNIST: Marginal categorical posterior of CP-VAE (**a**), INDq (**b**), INDp (**c**) and INDqp (**d**) with discrete latent variable with 150 categories.

**Figure 10:**
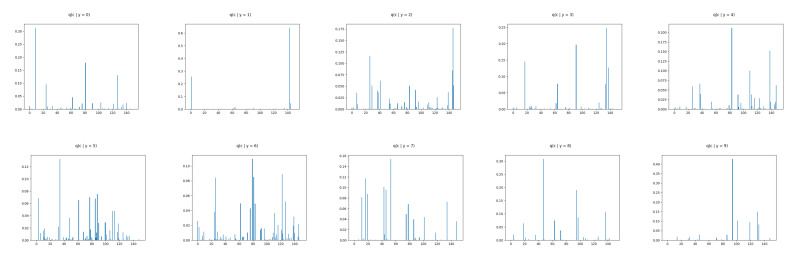
FashionMNIST: Marginal categorical posterior conditioned on each label of CP-VAE with discrete latent variable with 150 categories.

**Figure 11:**
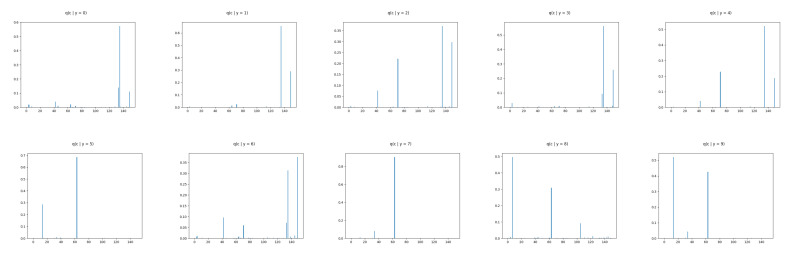
FashionMNIST: marginal categorical posterior conditioned on each label of INDq with discrete latent variable with 150 categories.

We show (Figure 6) that the learned discrete encoding in our model accurately captures the main source of variation of the data without any supervision. The unsupervised categories achieved by the model through the learned conditional prior correspond well to what a human annotator would do. Not only there are ten main categories (e.g., dresses, sandals), but our model also discovers subcategories among each main category (e.g., long and short sleeve dresses, hight heel, and flat sandals). In contrast, INDq (Figure 7) does not always capture the categories that generate a mix of images. This difference in the two models is because of two reasons. Firstly, our model learns the categorical posterior q(c) with many more categories having non-zero probability ($q(c_{k}\to 0)$) compared to the INDq, Figure 9. Secondly, our model also assigns high probability to different categories for each label, while, in INDq, some categories are assigned with high probability for more than one label, Figure 10 and Figure 11. Although INDp and INDqp are able to generate decent samples, Figure 8, none of them are able to accurately capture the categories of the data, confirming our theoretical analysis in Section 5.1.

Figure 9 illustrates the categorical marginal posterior of our model, INDq, INDp, and INDqp models. As we can see in Figure 9b, in INDq the vast majority of the categories of the discrete latent variable have very low probability nearing zero. This indicates that the model learns the distributions over a small numbers of categories making it almost impossible to generate samples from different subgroups. In contrast, our model not only learns the distributions over many more categories of the discrete latent variable, Figure 9a, but also the majority of them has probability higher than 1/150. For the INDp and INDqp models, the majority of the categories are used, Figure 9c,d, resulting an almost uniform marginal categorical posterior.

**Figure 12:**
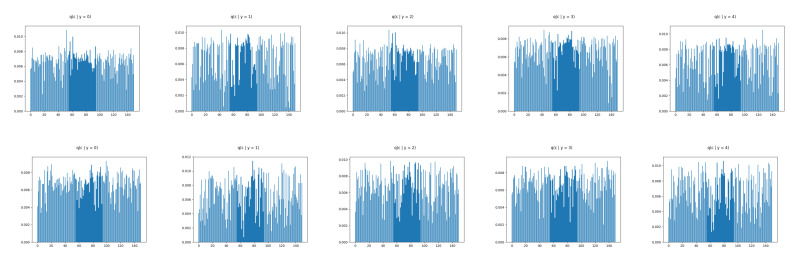
FashionMNIST: Marginal categorical posterior conditioned on the 5 first labels of INDp (1st row) and INDqp (2nd row) with discrete latent variable with 150 categories.

The discrete latent variables seems to discover the true labels in an unsupervised manner as the major source of variability and therefore we confirm this by examining the conditional marginal categorical posteriors. This is implemented by training our model without any supervision and, at the end, we use the the true labels to compute the marginal categorical posterior condition on each label. In Figure 10 and Table 2, we can see that our model uses with high probability different categories of the discrete latent variable for each label. This makes it feasible to generate new images conditioned on each label avoiding mix image generations. Moreover, our model for each label learns more than one category with high probability allowing to capture the different subgroups among the labels. In contrast, INDq, INDp and INDqp models, Figure 11 and Figure 12, Table 2, use the same categories of the discrete latent variable in more than one label, resulting in a mix of images.

**Figure 13:**
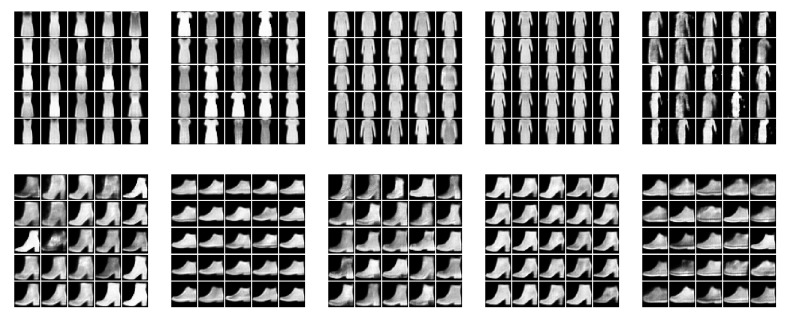
New variations of label specific individual FashionMNIST generated by CP-VAE model with a latent discrete variable with 150 categories. Samples in the same row belong to the same class label and samples in the same subplot were generated from the same discrete category.

If the true class label is available at the training data, then CP-VAE is also able to generate samples from specific labels. This can be done by computing the marginal categorical posterior for each class, q(c|x∈classi) and then for each class fixing the discrete latent variable to the categories with the highest probabilities and sampling the continuous latent from the corresponding learned priors. In this way, we can generate samples form a specific label, but we can also generate samples from different subcategories of this label, by conditioning on different categories $c_{k}$ Figure 13. This is just a theoretical exercise meant to show the power of our model. If the labels were truly available, they should be better used for training in a supervised manner. However, this is not the setting that we consider in the unsupervised learning problem that our CP-VAE is developed for.

**Figure A1:**
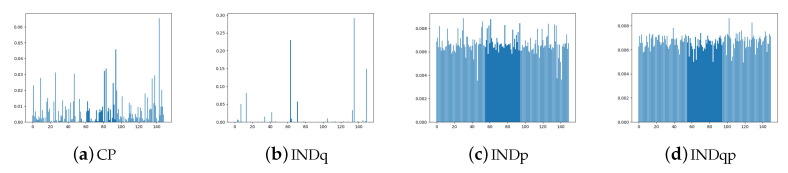
MINST: Marginal categorical posterior of CPVAE (**a**), INDq (**b**), INDp (**c**) and INDqp (**d**) with discrete latent variable with 150 categories.

**Figure A2:**
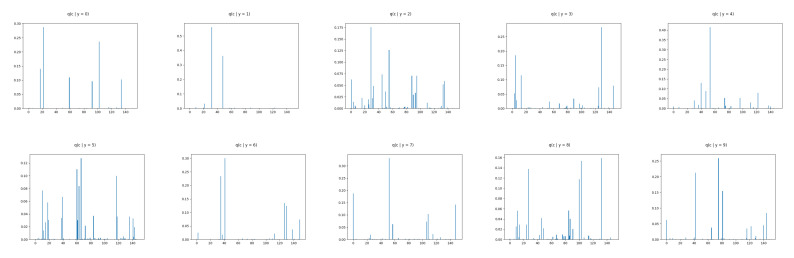
MINST: Marginal categorical posterior condition on each label of CPVAE with discrete latent variable with 150 categories.

**Figure A3:**
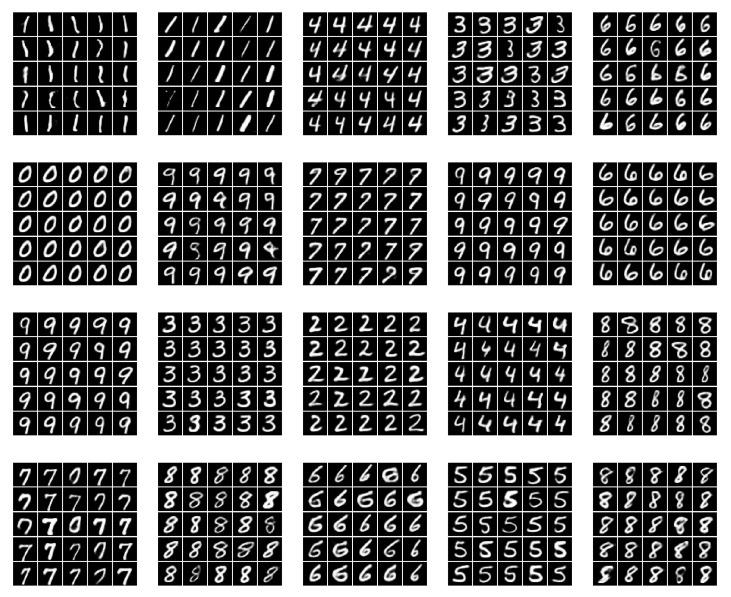
New variations of individual MNIST digits generated by our CP-VAE model with a latent discrete variable with 150 categories. Examples in the same subplot were generated from the same discrete category. To generate the samples we randomly use 20 categories with probability higher than 1/150.

**Figure A4:**
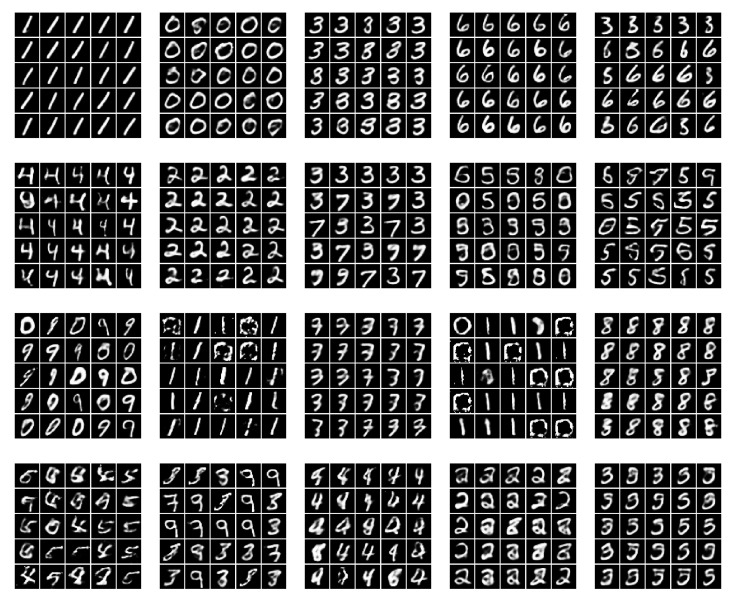
New variations of individual MNIST digits generated by INDq model with a latent discrete variable with 150 categories. Examples in the same subplot were generated from the same discrete category. To generate the samples we randomly use 20 categories with probability higher than 1/150.

**Figure A5:**
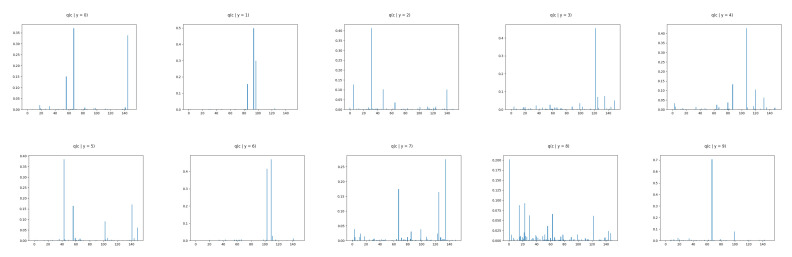
MINST: Marginal categorical posterior condition on each label of INDq with discrete latent variable with 150 categories.

**Figure A6:**
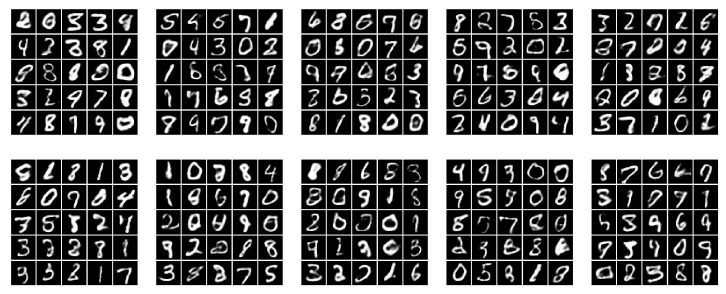
New variations of individual MNIST generated by INDp model (1st–2nd row) and INDqp model (3rd–4th row) with a latent discrete variable with 150 categories. Samples in the same subplot were generated from the same discrete category. For both models we randomly pick 10 categories categories with probability higher than 1/150 in order to generate the samples.

Repeating the analysis (for better flow of the text the corresponding Figures are left for the Appendix) using the MNIST data set we observe the same behaviour for our model. It uses the vast majority of the discrete latent variable with high probability allowing to discover a lot of different clusters among the data, Figure A1a in Appendix B. Furthermore, different categories are activated with high probability for each label allowing to discover important factors of data variations within each label, Figure A2 and Figure A3. The INDq model, due to the simplicity of MNIST dataset, uses more categories of the discrete latent variable with higher probability, Figure A1b, and discovers more subgroups as compared to FashionMNIST dataset. Generating samples using the 20 categories of the discrete latent variable with the highest probability, Figure A4, we can see that the model is able to generate few samples from different subgroups but also generates mix of images for most subplots. This can also be confirmed from the marginal categorical posterior conditioned on each label, Figure A5. There are a few categories that are used only in one label, resulting in samples only from a specific subgroup, while some categories appear in more than one label with high probability, causing a generation of mixed images. The INDp and INDqp models are not able to capture the possible underlying clusters of the data, even in this relatively simple dataset Figure A6.

Repeating the analysis (for better flow of the text the corresponding Figures are left for the Appendix) using the MNIST data set we observe the same behaviour for our model. It uses the vast majority of the discrete latent variable with high probability allowing to discover a lot of different clusters among the data, Figure A1a in Appendix B. Furthermore, different categories are activated with high probability for each label allowing to discover important factors of data variations within each label, Figure A2 and Figure A3. The INDq model, due to the simplicity of MNIST dataset, uses more categories of the discrete latent variable with higher probability, Figure A1b, and discovers more subgroups as compared to FashionMNIST dataset. Generating samples using the 20 categories of the discrete latent variable with the highest probability, Figure A4, we can see that the model is able to generate few samples from different subgroups but also generates mix of images for most subplots. This can also be confirmed from the marginal categorical posterior conditioned on each label, Figure A5. There are a few categories that are used only in one label, resulting in samples only from a specific subgroup, while some categories appear in more than one label with high probability, causing a generation of mixed images. The INDp and INDqp models are not able to capture the possible underlying clusters of the data, even in this relatively simple dataset Figure A6.

**Figure A7:**
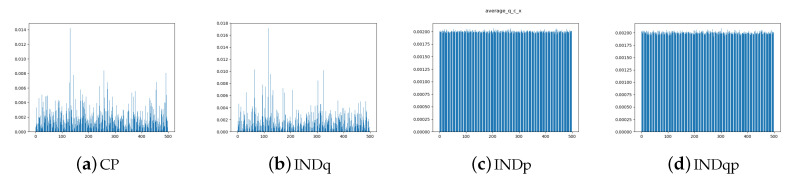
Omniglot: Marginal categorical posterior of CPVAE (**a**), INDq (**b**), INDp (**c**) and INDqp (**d**) with discrete latent variable with 500 categories.

**Figure A8:**
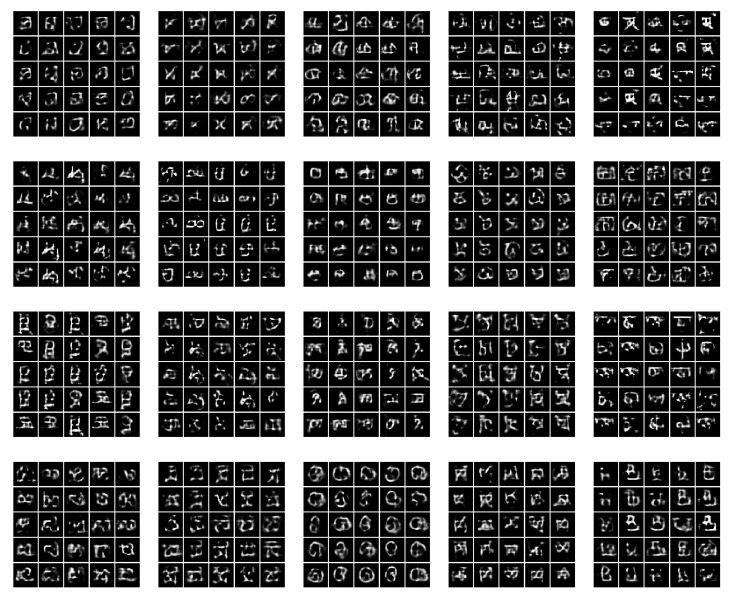
New variations of individual Omniglot symbols generated by our CP-VAE model with a latent discrete variable with 500 categories. Examples in the same subplot were generated from the same discrete category. To generate the samples we randomly use 20 categories with probability higher than 1/500.

**Figure A9:**
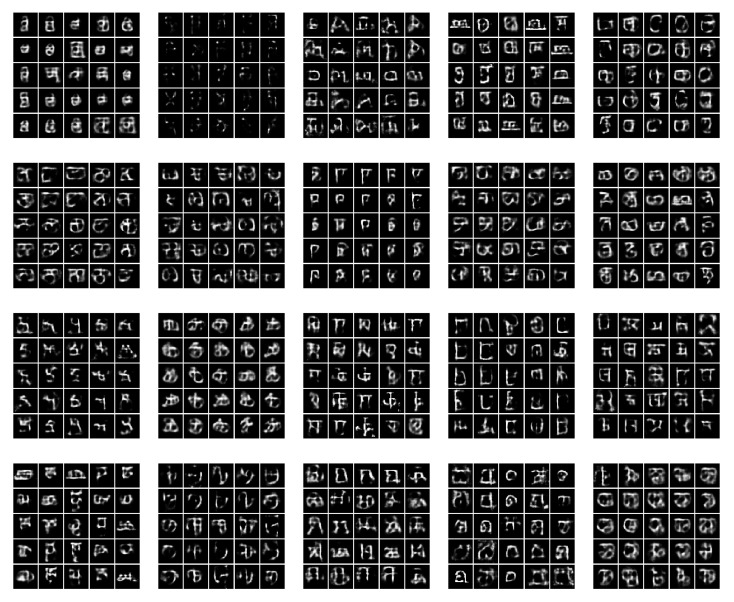
New variations of individual Omniglot symbols generated by INDq model with a latent discrete variable with 500 categories. Examples in the same subplot were generated from the same discrete category. To generate the samples we randomly use 20 categories with probability higher than 1/500.

**Figure A10:**
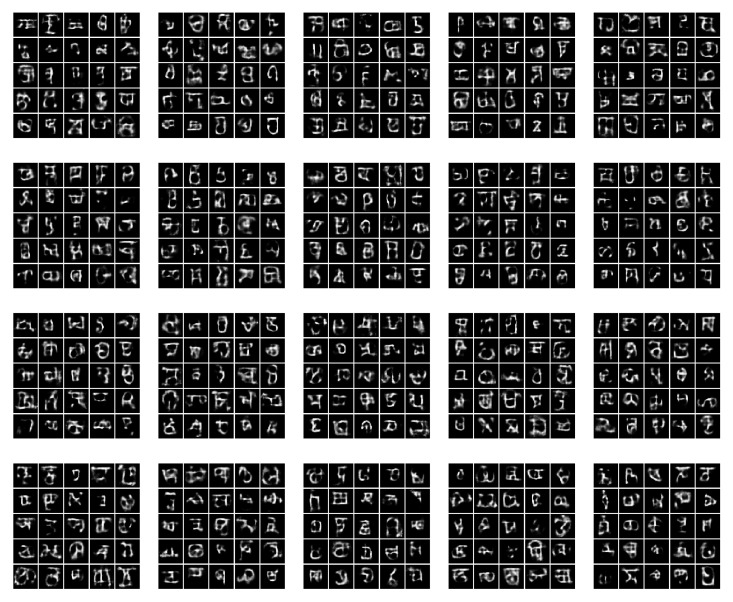
New variations of individual Omniglot symbols generated by INDp model (1st–2nd row) and INDqp model (3rd–4rth row) with a latent discrete variable with 500 categories. Examples in the same subplot were generated from the same discrete category. To generate the samples we randomly use 10 categories with probability higher than 1/500.

Unlike MNIST and FashionMNIST, which have a small number of labels with many images of each label and a large amount of data, the Omniglot dataset [37] consists of 105 × 105 binary images across 1628 labels with only 20 images per label. This data set allows for demonstrating that our model is able to capture some structure of the data even in regimes with limited amounts of data within a big number of categories. Our model uses the vast majority of the discrete latent variable with high probability allowing to discover a lot of different clusters among the data, Figure A7a. As Figure A8 illustrates, our model seems to recognise the modes over the original data and it is able to conditionally generate new samples from the underlying multi-modal distribution even in this more challenging dataset. INDq seems also able to discover some structure, Figure A9, but again it mostly generates a mix of images. As in the previous data sets the INDp and INDqp models are not able to capture the possible underlying clusters of the data, Figure A10 in Appendix C.

Finally, we compare the performance of our CP-VAE to a number of standard baselines varying also the size of the categorical variable. We experiment with {10,150,500} categories for MNIST and FashionMNIST and {50,500} categories for Omniglot. The methods that we compare to are the simple VAE (VAE), the three combinations of continuous and discrete latent variable models INDq, INDp, INDqp and the following methods from [17]: VAE with Mixture of Gaussians prior (MG), VAE with VampPrior (VP), hierarchical two-layerd VAE with VampPrior (HVP), and hierarchical two-layerd VAE with simple fixed prior (HVAE). For the VP and MG methods, we use the same numbers of pseudo-inputs and mixture components as the number of the latent categories. For the two layers models we use 40 latent variables at each layer.

We summarize the numerical results in terms of the negative variational lower bound calculated over the test data in Table 3. Our model achieves better results when compared to INDq model, the other method with learned prior, in all of the cases. The INDp and INDqp seem to perform slightly better and VampPrior and especially the hierarchical VampPrior method consistently perform the best. However, this numerical evaluation should be treated with care and considered in the context. As explained in Section 3, good values of the variational lower bound objective do not guarantee good generations and certainly not good control over the distributional clusters, which is the goal of our CP-VAE.

**Figure 14:**
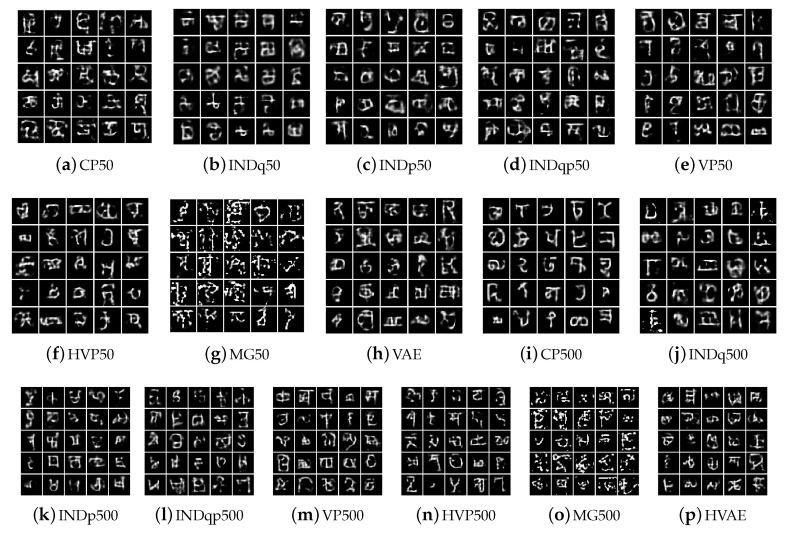
New data examples from the Omniglot dataset generated by the various methods with increasing number of the prior components.

**Figure 15:**
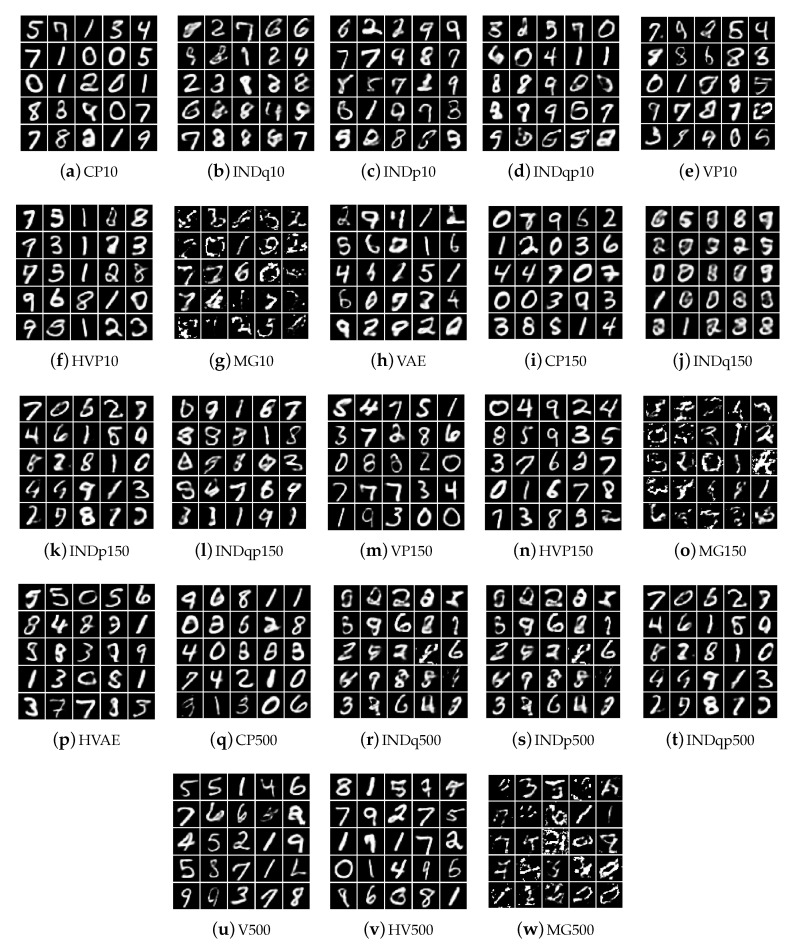
New data examples from the MNIST dataset generated by the various methods with increasing number of the prior components.

**Figure 16:**
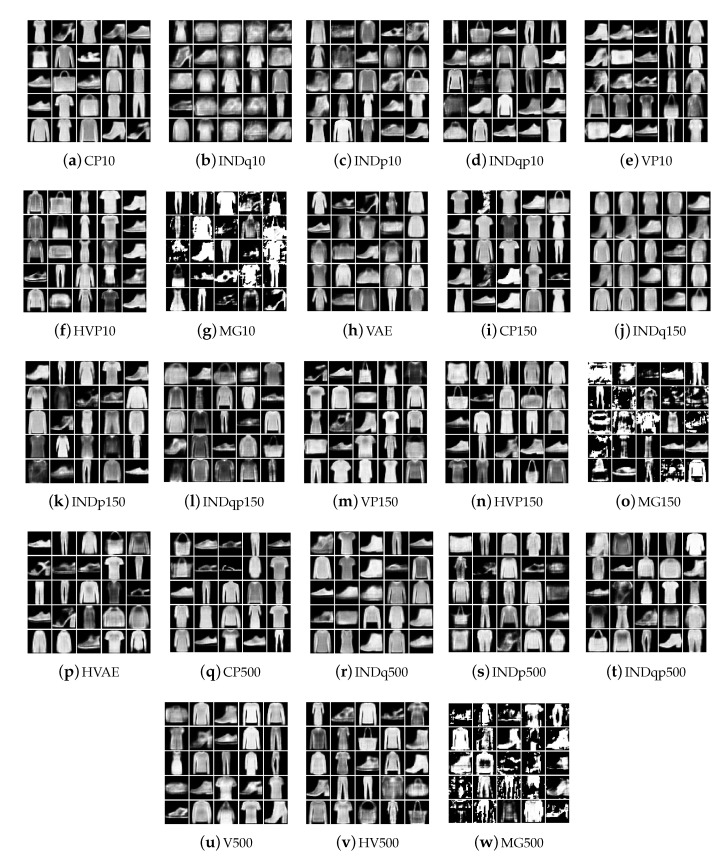
New data examples from the FashionMNIST dataset generated by the various methods with increasing number of the prior components.

We present the new data examples generated by the various methods in Figure 14, Figure 15 and Figure 16 for the Omniglot, MNIST, and FashionMNIST data, respectively. Our model is able to consistently generate good quality new samples for all of the datasets, irrespective of the number of latent categorical components. The other three combinations of continuous and discrete latent models that we examine (INDq, INDqp, and INDp) are also able to generate decent samples. However, as previously explained, our model has a critical advantage, since these cannot generate conditionally. The other methods (all VampPrior variations, including the two-layer hierarchical, and the MG) fail to generate quality examples with only 10 components within the prior. They also seem to collapse to generating examples only from a few digits (items, symbols), which suggest an important lack of flexibility available for the generations. As the number of components (pseudo-inputs) in the prior mixture increases, the VampPrior generations tend to improve, with the hierarchical version of the method systematically outperforming the simple VP version.

## 7. Conclusions

In this paper, we introduce CP-VAE, an unsupervised generative model that is able to learn the multi-modal probabilistic structure of the data. We propose a conditionally structured latent representation that enables our model to discover the modes in the training data distribution. This is achieved by decomposing the latent representation into a continuous and a discrete component and through a better matching between prior and posterior distributions by jointly learning their parameters from the data. The experimental results demonstrate that our approach is able to recover the modes over the original data in an unsupervised manner with a performance similar to that of a human annotator and that CP-VAE is able to conditionally generate new samples from the individual modes of the underlying distribution. In addition, we conduct a theoretical and experimental analysis of various independence assumptions on the continuous and discrete latent representations adopted in the related literature and argue in favour of our more general model formulation.

## Figures and Tables

**Table 1:** Independence assumptions for discrete-continuous latent variable models and the corresponding decomposition of the *B* and *C* terms in Equation (19).

Model	$q_{\phi }\left(z,c|x\right)$	$p_{\varphi }\left(z,c\right)$	B1	C1	Refs.
CP-VAE	$q_{\phi }\left(z|x,c\right)q_{\phi }\left(c|x\right)$	$p_{\varphi }\left(z|c\right)p\left(c\right)$	$I_{q}\left(z|c,x|c\right)$	$E_{q(c)}\left[\mathrm{KL}\left(q_{\phi }\left(z|c\right)∥p_{\varphi }\left(z|c\right)\right)\right]$	
INDq	$q_{\phi }\left(z|x\right)q_{\phi }\left(c|x\right)$	$p_{\varphi }\left(z|c\right)p\left(c\right)$	$E_{q_{\phi }\left(c,x\right)}[\mathrm{KL}\left(q_{\phi }\left(z|x\right)∥q_{\phi }\left(z|c\right)\right]$	$E_{q(c)}\left[\mathrm{KL}\left(q_{\phi }\left(z|c\right)∥p_{\varphi }\left(z|c\right)\right)\right]$	[7]
INDp	$q_{\phi }\left(z|x,c\right)q_{\phi }\left(c|x\right)$	p(z)p(c)	$I_{q}\left(z,\left(c,x\right)\right)$	$\mathrm{KL}(q_{\phi }\left(z\right)∥p\left(z\right))$	[26,34]
INDqp	$q_{\phi }\left(z|x\right)q_{\phi }\left(c|x\right)$	p(z)p(c)	$I_{q}\left(z,x\right)$	$\mathrm{KL}(q_{\phi }\left(z\right)∥p\left(z\right))$	[13,21]

**Table 2:** FashionMNIST: first, five categories with higher probability for each label based on the marginal categorical posterior condition on each label of CPVAE and INDq with discrete latent variable with 150 categories. With bold, we mark the categories that appear in more than one label.

	CPVAE					INDq				
label 0	9	80	127	24	62	135	133	149	42	64
label 1	143	1	144	64	135	135	149	71	64	3
label 2	146	25	145	41	29	135	149	71	42	3
label 3	135	91	17	138	64	135	149	133	3	148
label 4	83	138	109	37	147	135	71	149	42	114
label 5	34	88	77	3	85	63	13	34	39	31
label 6	79	122	81	26	127	149	135	42	133	71
label 7	53	16	43	46	19	63	34	13	31	126
label 8	47	95	137	97	63	7	63	105	123	145
label 9	94	130	101	119	132	13	63	34	31	145

**Table 3:** Comparison of negative variational lower bounds for the different methods over the test data sets.

	MNIST			FashionMNIST			Omniglot	
	c=10	c=150	c=500	c=10	c=150	c=500	c=50	c=500
CP	87.15	88.53	89.91	232.52	233.79	234.89	117.51	120.64
INDq	89.67	92.15	93.38	232.71	234.41	234.93	125.28	124.48
INDp	88.20	88.77	88.53	229.83	230.41	231.34	120.92	121.83
INDqp	87.93	88.21	88.98	228.65	230.98	231.18	119.99	120.82
VAE	88.75	—	—	231.49	—	—	115.06	—
MG	89.43	88.96	88.85	267.07	272.60	274.55	116.31	116.12
VP	87.94	86.55	86.07	230.87	229.82	270.83	114.01	113.74
HVAE	86.7	—	—	230.10	—	—	110.81	—
HVP	85.90	85.09	85.01	229.67	229.36	229.62	110.50	110.16

## Appendix A. Proofs

### Appendix A.1. Proofs of Section 4

**Proof** **of** **Equation (12).**

$$\begin{array}{cc} \underset{B}{\underset{︸}{\mathrm{KL}\left(q_{\phi }\left(z,c|x\right)∥p_{\varphi }\left(z,c\right)\right)}}\; & =E_{q_{\phi }\left(z|x,c\right)q_{\phi }\left(c|x\right)}\log\frac{q_{\phi }\left(c|x\right)}{p(c)}\frac{q_{\phi }\left(z|x,c\right)}{p_{\varphi }\left(z|c\right)}\\ \; & =\underset{B1}{\underset{︸}{\mathrm{KL}\left(q_{\phi }\left(c|x\right)∥p\left(c\right)\right)}}+\underset{B2}{\underset{︸}{E_{q_{\phi }\left(c|x\right)}\mathrm{KL}\left(q_{\phi }\left(z|x,c\right)∥p_{\varphi }\left(z|c\right)\right)}} \end{array}$$

□

**Proof** **of** **Equation (13).**

$$\begin{array}{cc} \underset{B1}{\underset{︸}{\mathrm{KL}\left(q_{\phi }\left(c|x\right)\left|\right|p\left(c\right)\right)}} & =E_{q_{\phi }\left(c|x\right)}\left[\log q_{\phi }\left(c|x\right)-\log K^{-1}\right]=-H\left(q_{\phi }\left(c|x\right)\right)+\log K \end{array}$$

□

### Appendix A.2. Proofs of Section 5

**Proof** **of** **Equation (17).**

$$\begin{array}{cc} E_{p(x)} & \mathrm{KL}\left(q_{\phi }\left(z,c|x\right)∥p_{\varphi }\left(z,c\right)\right)\\ \; & =E_{p(x)}\mathrm{KL}\left(q_{\phi }\left(c|x\right)∥p\left(c\right)\right)+E_{p(x)}E_{q_{\phi }\left(c|x\right)}\mathrm{KL}\left(q_{\phi }\left(z|x,c\right)∥p_{\varphi }\left(z|c\right)\right)\\ \; & =E_{q_{\phi }\left(x,c\right)}\left[\log\frac{q_{\phi }\left(c|x\right)}{q_{\phi }\left(c\right)}+\log\frac{q_{\phi }\left(c\right)}{p(c)}\right]+E_{q_{\phi }\left(x,z,c\right)}\left[\log\frac{q_{\phi }\left(z,c|x\right)}{q_{\phi }\left(c|x\right)q_{\phi }\left(z|c\right)}+\log\frac{q_{\phi }\left(z|c\right)}{p_{\varphi }\left(z|c\right)}\right]\\ \; & =\mathrm{KL}\left(q_{\phi }\left(c\right)∥p\left(c\right)\right)+E_{q_{\phi }\left(c\right)}\mathrm{KL}\left(q_{\phi }\left(z|c\right)∥p_{\varphi }\left(z|c\right)\right)+E_{q_{\phi }\left(x,z,c\right)}\log\frac{q_{\phi }\left(z,c|x\right)}{q_{\phi }\left(z|c\right)q_{\phi }\left(c\right)}\\ \; & =\log K-H\left(q_{\phi }\left(c\right)\right)+E_{q_{\phi }\left(c\right)}\mathrm{KL}\left(q_{\phi }\left(z|c\right)∥p_{\varphi }\left(z|c\right)\right)+E_{q_{\phi }\left(x,z,c\right)}\log\frac{q_{\phi }\left(z,c|x\right)}{q_{\phi }\left(z,c\right)}\\ \; & =\log K-H\left(q_{\phi }\left(c\right)\right)+E_{q_{\phi }\left(c\right)}\mathrm{KL}\left(q_{\phi }\left(z|c\right)∥p_{\varphi }\left(z|c\right)\right)+I_{q}\left((z,c),x\right) \end{array}$$

□

**Proof** **of** **Equation (19).**

$$\begin{array}{cc} L(\theta ,\phi )\; & =E_{q_{\phi }\left(z,c,x\right)}\left[\log\frac{p_{\theta }\left(x,z,c\right)}{q_{\phi }\left(z,c|x\right)}+\log\frac{p_{\varphi }\left(z,c\right)}{p_{\varphi }\left(z,c\right)}+\log\frac{q_{\phi }\left(z,c\right)}{q_{\phi }\left(z,c\right)}\right]\\ \; & =E_{q_{\phi }\left(z,c,x\right)}\left[\log\frac{p_{\theta }\left(x,z,c\right)}{p_{\varphi }\left(z,c\right)}\right]-E_{q_{\phi }\left(z,c,x\right)}\left[\log\frac{q_{\phi }\left(z,c|x\right)}{q_{\phi }\left(z,c\right)}\right]-E_{q_{\phi }\left(z,c,x\right)}\left[\log\frac{q_{\phi }\left(z,c\right)}{p_{\varphi }\left(z,c\right)}\right]\\ \; & =\underset{A}{\underset{︸}{E_{q_{\phi }\left(z,c,x\right)}\left[\log p_{\theta }\left(x|z,c\right)\right]}}-\underset{B}{\underset{︸}{I_{q}\left((z,c),x)\right)}}-\underset{C}{\underset{︸}{\mathrm{KL}(q_{\phi }\left(z,c\right)∥p_{\varphi }\left(z,c\right))}} \end{array}$$

□

The staring point for all the models is Equation (19). Here we decompose the terms *B* and *C* as per the independence assumptions listed in Table 1.

**Proof** **of** **CP-VAE objective:**

$$\begin{array}{cc} B:\;I_{q}\left(\left(z,c\right),x\right)\; & =E_{q_{\phi }\left(z,c,x\right)}\left[\log\frac{q_{\phi }\left(z,c|x\right)}{q_{\phi }\left(z,c\right)}\right]\\ \; & =E_{q_{\phi }\left(z,c,x\right)}\left[\log\frac{q_{\phi }\left(z|x,c\right)q_{\phi }\left(c|x\right)}{q_{\phi }\left(z|c\right)q_{\phi }\left(c\right)}+\log\frac{q_{\phi }\left(x|c\right)}{q_{\phi }\left(x|c\right)}\right]\\ \; & =E_{q_{\phi }\left(z,c,x\right)}\left[\log\frac{q_{\phi }\left(z,x|c\right)}{q_{\phi }\left(z|c\right)q_{\phi }\left(x|c\right)}\right]+E_{q_{\phi }\left(z,c,x\right)}\left[\log\frac{q_{\phi }\left(c|x\right)}{q_{\phi }\left(c\right)}\right]\\ \; & =I_{q}\left(z|c,x|c\right)+I_{q}\left(c,x\right)\\ \; & =B1+B2 \end{array}$$

$$\begin{array}{cc} C:\;\mathrm{KL}(q_{\phi }\left(z,c\right)∥p_{\varphi }\left(z,c\right))\; & =\mathrm{KL}(q_{\phi }\left(z|c\right)q_{\phi }\left(c\right)∥p_{\varphi }\left(z|c\right)p\left(c\right))\\ \; & =E_{q_{\phi }\left(z|c\right)q_{\phi }\left(c\right)}\left[\log\frac{q_{\phi }\left(z|c\right)}{p_{\varphi }\left(z|c\right)}\right]+E_{\log q_{\phi }\left(z|c\right)q_{\phi }\left(c\right)}\left[\frac{q_{\phi }\left(c\right)}{p(c)}\right]\\ \; & =E_{q_{\phi }\left(c\right)}\left[\mathrm{KL}\left(q_{\phi }\left(z|c\right)∥p_{\varphi }\left(z|c\right)\right)\right]+\mathrm{KL}\left(q_{\phi }\left(c\right)∥p\left(c\right)\right)\\ \; & =C1+C2 \end{array}$$

□

**Proof** **of ** **INDq objective:**

$$\begin{array}{cc} B:\;I_{q}\left(\left(z,c\right),x\right)\; & =E_{q_{\phi }\left(z,c,x\right)}\left[\log\frac{q_{\phi }\left(z,c|x\right)}{q_{\phi }\left(z,c\right)}\right]\\ \; & =E_{q_{\phi }\left(z,c,x\right)}\left[\log\frac{q_{\phi }\left(z|x\right)q_{\phi }\left(c|x\right)}{q_{\phi }\left(z|c\right)q_{\phi }\left(c\right)}\right]\\ \; & =E_{q_{\phi }\left(x,c\right)q_{\phi }\left(z|x\right)}\left[\log\frac{q_{\phi }\left(z|x\right)}{q_{\phi }\left(z|c\right)}\right]+E_{q_{\phi }\left(z,c,x\right)}\left[\log\frac{q_{\phi }\left(c|x\right)}{q_{\phi }\left(c\right)}\right]\\ \; & =E_{q_{\phi }\left(x,c\right)}\mathrm{KL}\left(q_{\phi }\left(z|x\right)\right)∥q_{\phi }\left(z|c\right))+I_{q}\left(c,x\right)\\ \; & =B1+B2 \end{array}$$

*C* term is the same as in CP-VAE.□

**Proof** **of ** **INDp objective:**

$$\begin{array}{cc} B:\;I_{q}\left(\left(z,c\right),x\right)\; & =E_{q_{\phi }\left(z,c,x\right)}\left[\log\frac{q_{\phi }\left(z,c|x\right)}{q_{\phi }\left(z,c\right)}\right]\\ \; & =E_{q_{\phi }\left(z,c,x\right)}\left[\log\frac{q_{\phi }\left(z|x,c\right)q_{\phi }\left(c|x\right)}{q_{\phi }\left(z\right)q_{\phi }\left(c\right)}+\log\frac{q_{\phi }\left(x,c\right)}{q_{\phi }\left(x,c\right)}\right]\\ \; & =E_{q_{\phi }\left(z,c,x\right)}\left[\log\frac{q_{\phi }\left(z,c,x\right)}{q_{\phi }\left(z\right)q_{\phi }\left(x,c\right)}\right]+E_{q_{\phi }\left(z,c,x\right)}\left[\log\frac{q_{\phi }\left(c|x\right)}{q_{\phi }\left(c\right)}\right]\\ \; & =I_{q}\left(z,\left(x,c\right)\right)+I_{q}\left(c,x\right) \end{array}$$

$$\begin{array}{cc} C:\;\mathrm{KL}(q_{\phi }\left(z,c\right)∥p_{\varphi }\left(z,c\right))\; & =\mathrm{KL}(q_{\phi }\left(z|c\right)q_{\phi }\left(c\right)∥p_{\varphi }\left(z|c\right)p\left(c\right))\\ \; & =E_{q_{\phi }\left(z,c,x\right)}\left[\log\frac{q_{\phi }\left(z|x,c\right)q_{\phi }\left(c|x\right)}{q_{\phi }\left(z\right)q_{\phi }\left(c\right)}+\log\frac{q_{\phi }\left(x,c\right)}{q_{\phi }\left(x,c\right)}\right]\\ \; & =E_{q_{\phi }\left(z,c,x\right)}\left[\log\frac{q_{\phi }\left(z,c,x\right)}{q_{\phi }\left(z\right)q_{\phi }\left(x,c\right)}\right]+E_{q_{\phi }\left(z,c,x\right)}\left[\log\frac{q_{\phi }\left(c|x\right)}{q_{\phi }\left(c\right)}\right]\\ \; & =\mathrm{KL}\left(q_{\phi }\left(z\right)∥p\left(z\right)\right)+\mathrm{KL}\left(q_{\phi }\left(c\right)∥p\left(c\right)\right)\\ \; & =C1+C2 \end{array}$$

□

**Proof** **of ** **INDqp objective:**

$$\begin{array}{ccc} B:\;I_{q}\left(\left(z,c\right),x\right)\; & =E_{q_{\phi }\left(z,c,x\right)}\left[\log\frac{q_{\phi }\left(z,c|x\right)}{q_{\phi }\left(z,c\right)}\right]\\ \; & =E_{q_{\phi }\left(z,c,x\right)}\left[\log\frac{q_{\phi }\left(z|x\right)q_{\phi }\left(c|x\right)}{q_{\phi }\left(z\right)q_{\phi }\left(c\right)}\right]\\ \; & =E_{q_{\phi }\left(z,c,x\right)}\left[\log\frac{q_{\phi }\left(z|x\right)}{q_{\phi }\left(z\right)}\right]+E_{q_{\phi }\left(z,c,x\right)}\left[\log\frac{q_{\phi }\left(c|x\right)}{q_{\phi }\left(c\right)}\right]\\ \; & =I_{q}\left(z,x\right)+I_{q}\left(c,x\right)\; & =B1+B2 \end{array}$$

*C* term is the same as in INDp model.□

### Appendix A.3. Proofs of Section 5.2

**Proof.** $E_{p(x)}\left[KL(q_{\phi }\left(z|x\right)∥p\left(z\right))\right]=I_{q}\left(z,x\right)+\mathrm{KL}\left(q_{\phi }\left(z\right)∥p\left(z\right)\right)\;:$

$$\begin{array}{cc} E_{p(x)}\left[\mathrm{KL}(q_{\phi }\left(z|x\right)∥p\left(z\right))\right]\; & =E_{q_{\phi }\left(z,x\right)}\left[\log\frac{q_{\phi }\left(z|x\right)}{p(z)}\right]\\ \; & =E_{q_{\phi }\left(z,x\right)}\left[\log\frac{q_{\phi }\left(z|x\right)}{p(z)}\frac{q_{\phi }\left(z\right)}{q_{\phi }\left(z\right)}\right]\\ \; & =E_{q_{\phi }\left(z,x\right)}\left[\log\frac{q_{\phi }\left(z|x\right)}{q_{\phi }\left(z\right)}\right]+E_{q_{\phi }\left(z\right)}\left[\log\frac{q_{\phi }\left(z\right)}{p(z)}\right]\\ \; & =I_{q}\left(z,x\right)+\mathrm{KL}\left(q_{\phi }\left(z\right)∥p\left(z\right)\right) \end{array}$$

□

### Appendix A.4. Maximizing the Negative RE is Equivalent to Maximizing a Lower Bound on MI between the Latent Variables (***z***,***c***) and ***x***

**Proof.** The non-negativity of the KL divergence implies:

(A1) $$\begin{array}{cc} \mathrm{KL}(q_{\phi }\left(x|z,c\right) & ∥p\left(x|z,c\right))=E_{\log q_{\phi }\left(x|z,c\right)}\left[\log p\left(x|z,c\right)\right]-E_{\log q_{\phi }\left(x|z,c\right)}\left[\log q_{\phi }\left(x|z,c\right)\right]\geq 0\\ \; & \Rightarrow E_{\log q_{\phi }\left(x|z,c\right)}\left[\log q_{\phi }\left(x|z,c\right)\right]\geq E_{\log q_{\phi }\left(x|z,c\right)}\left[\log p\left(x|z,c\right)\right] \end{array}$$

This leads to a lower bound on the mutual Information $I_{q}\left(\left(z,c\right),x\right)$

(A2) $$\begin{array}{cc} I_{q}\left(\left(z,c\right),x\right) & =\mathrm{KL}(q_{\phi }\left(x,z,c\right)∥p_{D}\left(x\right)q_{\phi }\left(z,c\right))\\ \; & =E_{\log q_{\phi }\left(x,z,c\right)}\left[\log q_{\phi }\left(x|z,c\right)\right]-E_{\log q_{\phi }\left(x,z,c\right)}\left[p_{D}\left(x\right)\right]\\ \; & =E_{\log q_{\phi }\left(x,z,c\right)}\left[\log q_{\phi }\left(x|z,c\right)\right]+H\left(p_{D}\left(x\right)\right)\\ \; & =E_{\log q_{\phi }\left(x,z,c\right)}\left[\log q_{\phi }\left(x|z,c\right)\right]+H\left(p_{D}\left(x\right)\right)+\mathrm{KL}\left(q_{\phi }\left(x|z,c\right)∥p\left(x|z,c\right)\right)\\ \; & \geq E_{\log q_{\phi }\left(x,z,c\right)}\left[\log p\left(x|z,c\right)\right]+H\left(p_{D}\left(x\right)\right) \end{array}$$

where the first term is the negative reconstruction error and the second, H(p(x)) is the entropy. This means that maximizing the negative reconstruction error we maximize a lower bound on $I_{q}\left(\left(z,c\right),x\right)$. □

## Appendix B. MNIST

Results from training CP-VAE, INDq, INDp and INDqp over the MNIST [35] data with 150 categories in the discrete latent variable (Table A1).

**Table A1:** MNIST: First five categories with higher probability for each label based on the marginal categorical posterior condition on each label of CPVAE and INDq with discrete latent variable with 150 categories. With bold we mark the categories that appear in more than one labels.

	CPVAE					INDq				
label 0	22	102	17	59	134	67	145	56	18	32
label 1	32	48	21	9	20	94	97	85	124	67
label 2	29	55	45	95	88	31	5	48	140	65
label 3	129	5	13	146	125	122	135	125	149	99
label 4	53	40	47	122	74	107	87	120	132	80
label 5	66	60	117	63	10	43	141	56	102	149
label 6	41	35	127	130	149	109	103	111	141	98
label 7	52	0	148	108	106	135	67	125	2	99
label 8	132	103	27	100	85	1	23	15	63	40
label 9	75	42	81	144	0	67	99	18	34	79

## Appendix C. Omniglot

Results from training CP-VAE, INDq, INDp and INDqp over the Omniglot [37] data with 500 categories in the discrete latent variable.
